# Pan‐cancer analysis reveals sex‐specific signatures in the tumor microenvironment

**DOI:** 10.1002/1878-0261.13203

**Published:** 2022-03-12

**Authors:** Junwei Han, Yang Yang, Xiangmei Li, Jiashuo Wu, Yuqi Sheng, Jiayue Qiu, Qian Wang, Ji Li, Yalan He, Liang Cheng, Yan Zhang

**Affiliations:** ^1^ 34707 College of Bioinformatics Science and Technology Harbin Medical University China; ^2^ 47822 School of Life Science and Technology Computational Biology Research Center Harbin Institute of Technology China

**Keywords:** immune and stromal scores, sex differences, sex‐specific prognostic biomarkers, tumor microenvironment, tumor mutational burden

## Abstract

The processes of cancer initiation, progression, and response to therapy are affected by the sex of cancer patients. Immunotherapy responses largely depend on the tumor microenvironment (TME), but how sex may shape some TME features, remains unknown. Here, we analyzed immune infiltration signatures across 19 cancer types from 1771 male and 1137 female patients in The Cancer Genome Atlas to evaluate how sex may affect the tumor mutational burden (TMB), immune scores, stromal scores, tumor purity, immune cells, immune checkpoint genes, and functional pathways in the TME. Pan‐cancer analyses showed higher TMB and tumor purity scores, as well as lower immune and stromal scores in male patients as compared to female patients. Lung adenocarcinoma, lung squamous carcinoma, kidney papillary carcinoma, and head and neck squamous carcinoma showed the most significant sex biases in terms of infiltrating immune cells, immune checkpoint gene expression, and functional pathways. We further focused on lung adenocarcinoma samples in order to identify and validate sex‐specific immune cell biomarkers with prognostic potential. Overall, sex may affect the tumor microenvironment, and sex‐specific TME biomarkers may help tailor cancer immunotherapy in certain cancer types.

## Graphical abstract

This study evaluated how sex may affect some TME features, including tumor mutational burden (TMB), immune scores, stromal scores, tumor purity, immune cells, immune checkpoint genes, and functional pathways in pan‐cancer and individual cancer types. The results revealed that sex may be a key factor affecting TME features in several cancer types.

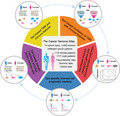

**Abbreviations**
AUC: area under the curve
BLCA: bladder urothelial carcinoma
COAD: colon adenocarcinoma
DC: dendritic cell
DLBC: diffuse large b‐cell lymphoma
GBM: glioblastoma multiforme
HNSC: head and neck squamous cell carcinoma
ICB: immune checkpoint blockade
iDC: interstitial dendritic cell
IL: interleukin
KIRC: kidney renal clear cell carcinoma
KIRP: kidney renal papillary cell carcinoma
LAML: acute myeloid leukemia
LGG: brain lower grade glioma
LIHC: liver hepatocellular carcinoma
LUAD: lung adenocarcinoma
LUSC: lung squamous cell carcinoma
NK: natural killer cell
PAAD: pancreatic adenocarcinoma
PD‐1: programmed cell death 1
pDC: plasmacytoid dendritic cell
READ: rectum adenocarcinoma
ROC: receiver operating characteristic
SARC: sarcoma
SKCM: skin cutaneous melanoma
ssGSEA: single‐sample Gene Set Enrichment Analysis
STAD: stomach adenocarcinoma
TCGA: The Cancer Genome Atlas
Tem: T effector memory cell
Tfh: follicular helper T cell
THCA: thyroid carcinoma
THYM: thymoma
TMB: tumor mutational burden
TME: tumor microenvironment
WRS: Wilcoxon rank‐sum test

## 1 Introduction

Sex differences in cancer initiation, progression, response to therapy, and prognosis outcome have been reported across multiple cancer types [1, 2]. It has been proposed that men show higher incidence and mortality in most cancer types. For example, the mortality of urinary bladder carcinomas in men clearly increases compared with women [3]. However, there are also some cancer types that undergo a higher risk in women than in men, for example, thyroid cancer [4]. Further, there are also significant differences in cancer immune responses between male and female patients in several cancer types, and women generally mount stronger innate and adaptive immune responses compared with men [5, 6]. Immune checkpoint blockade (ICB) therapies targeting programmed cell death 1 (PD‐1) or ligand 1 (PD‐L1) and cytotoxic T‐lymphocyte antigen‐4 (CTLA‐4) have demonstrated higher efficacy than standard therapies in several cancers [7]. Conforti et al. reported that men achieved greater efficacy from ICB therapies compared with women in randomized clinical trials including 11 351 patients with advanced or metastatic cancers [8]. This trend conforms to the situation that ICB therapies may block the immune inhibitory signals employed by tumor cells and then stimulate the body’s immune response; as women generally exhibit a stronger immune microenvironment, this may result in women receiving less therapy effect than men simply through enhancing immune response. However, Wallis et al. reported that there were no significant sex differences in terms of the efficacy of immunotherapy in 23 randomized clinical trials [9]. These conflicting results may be due to cancer patients of different sex possessing different TME and molecular features.

The TME contains many different non‐cancerous cell types in addition to cancer cells, such as fibroblasts and infiltrated immune cells, which play important roles in cancer progression, metastasis, and immune therapeutic efficacy [10, 11]. For example, it has been proposed that regulatory T cells (Tregs) and tumor‐associated macrophages were correlated with pro‐tumor functions [12, 13], B cells and natural killer (NK) cells have been shown variously positively or negatively to influence the prognosis of cancer patients [14]. Other immune cell types, such as CD8^+^ T cells, have been proposed to be associated with improved clinical outcomes and immunotherapy efficacy [15]. Recently, several computational techniques have been developed to estimate the relative abundance of different TME cells by using gene expression profiles of bulk tumors (from microarrays or RNA sequencing). For example, single sample gene set enrichment analysis (ssGSEA) uses cell‐type‐specific marker gene sets to infer the cell abundance by calculating the enrichment scores [16]. ImmuCellAI is designed to estimate the abundance of immune cells by integrating the ssGSEA and least‐square regression algorithms [17]. CIBERSORT quantifies cell fractions by applying the deconvolution algorithm to the signature matrices containing gene expression profiles of purified immune cells [18], and xCell integrates gene set enrichment with deconvolution approaches to enumerate the abundance of cell types [19]. These methods may help to investigate the association of TME cells with cancers and identify new immunotherapeutic biomarkers. Sex‐based differences in TME might also impact immune response; however, its contribution to cancer progression and prognosis across different cancer types has not been determined comprehensively [5].

Moreover, tumor mutational burden (TMB) was proposed to be correlated with TME in hepatocellular carcinoma and tracheal adenoid cystic carcinoma [20, 21]. TMB has also been shown to be correlated with response to PD‐1/PD‐L1 blockade in patients with diverse cancers, such as melanoma [22], non‐small cell lung cancer [23], and urothelial carcinoma [24]. Further, Li et al. discovered large differences in mutation burden between men and women in many but not all tumor types [25]. Wang et al. reported that TMB showed significant sex differences for the immune checkpoint inhibitors response, and its predictive power was significantly better for female than for male lung cancer patients [6]. As the tumor immune features may be different in men and women for some cancers, investigation of the sex differences in these features may help to identify sex‐specific immune signatures.

The availability of high‐throughput molecular data (e.g. whole‐exome sequencing, microarrays, RNA sequencing data) of multiple cancer types in The Cancer Genome Atlas (TCGA) data portal and the TME cell estimation methods based on bulk expression data provide an opportunity to analyze the immune features. Herein, we performed a comprehensive analysis to investigate sex‐based differences of cancer immune‐related features across a broad range of cancer types, including TMB, TME features (immune scores, stromal scores, tumor purity, and immune cells), somatic mutations, immune checkpoint genes, and immune functional pathways, to understand potential sex‐specific immune biomarkers and their effects on cancer progression, prognosis and, immunotherapy.

## 2 Materials and methods

### 2.1 Data acquisition and processing

We obtained all TCGA cancer patients except those with sex‐specific cancers (e.g. ovarian cancer, uterine cancer in women and prostate cancer, testicular cancer in men) from the GDC TCGA data portal (https://portal.gdc.cancer.gov/). The molecular data (e.g. somatic mutation, gene expression) and clinical characteristics (e.g. sex, age, tumor stage, and survival time) were downloaded accordingly. We retained the primary cancer samples for each cancer type, and samples younger than 18, older than 85 or lacking sex information were excluded from the analysis. For gene expression data, FPKM‐normalized profiles were used, and all expression values were then log2 (value + 1) transformed. Genes with > 90% of samples having zero expression values were removed from the respective cancer dataset. As our analyses attempt to test the sex differences in tumor microenvironment characterization, we focused on the samples with at least moderate immune infiltration to provide a fair comparison. To do this, we applied a deconvolution‐based CIBERSORT algorithm [18] to the gene expression profiles of each cancer to estimate the immune infiltration extent. The samples for each cancer dataset with an empirical CIBERSORT *P* < 0.05 were used for the following analysis. Finally, we required that each cancer type include sufficient sample sizes (≥ 10 for both male and female patients). Thus, we obtained 19 cancer types, including 2908 samples (1771 male and 1137 female samples) (Table S1). The above processing for sample filtering is shown in Fig. S1.

### 2.2 Tumor mutational burden

According to the Chalmers et al. study, TMB was defined as the number of somatic, coding, base substitution, and insertion or deletion mutations per megabase (Mb) of genome examined [26]. In our study, for each cancer type, we downloaded the somatic mutation annotation format (MAF) file (derived from Varscan2) from the GDC TCGA data portal, which is obtained from TCGA whole‐exome sequencing (WES) data. Based on the MAF file, we extracted non‐silent somatic mutations (nonsense, missense, splice‐site mutations, stop codon read‐throughs, change of start codon, frame‐shift indels, inframe indels) in the protein‐coding region of genes. For each sample, TMB was calculated as follows: N_mutation_/Length, where N_mutation_ is the number of non‐silent somatic mutations and Length is the length of coding regions (Mb). In TCGA, 38 Mb is generally used as the estimate of the length of coding regions; therefore, TMB was calculated for each sample.

### 2.3 Inference of the infiltration levels of immune cells and the scores of stromal and immune

Recently, a number of studies have applied single‐sample Gene Set Enrichment Analysis (ssGSEA) to infer the relative level of immune cell infiltration based on the cell‐specific signature from RNA profiling data [27]. In the study, we downloaded 24 immune cell type‐specific gene signatures from a Bindea et al. publication [28] (Table S2). Normalized gene expression data (log2‐transformed FPKM) were used to infer the relative tumor infiltration levels of 24 immune cell types using the ssGSEA algorithm. For each sample, the gene signatures of each immune cell were annotated to the ranked gene list based on the gene expression, and an enrichment score in the ssGSEA algorithm was used to represent the relative infiltration level of cell. We then applied the ESTIMATE (Estimation of STromal and Immune cells in MAlignant Tumor tissues using Expression data) algorithm [29] to infer stromal and immune scores of tumor samples, which reflect the level of infiltrating stromal and immune cells. The ESTIMATE algorithm which performs the ssGSEA strategy to infer these scores based on gene expression data, and its predictive ability has been validated in large and independent datasets (e.g. TCGA). Moreover, tumor purity, which is the proportion of cancer cells in the admixture, has been found to be significantly associated with clinical features and tumor biology. The tumor purity of each tumor sample was then inferred by using the ESTIMATE algorithm by combining the stromal and immune scores.

### 2.4 Sex differences analysis of tumor microenvironment features

The Wilcoxon rank‐sum (WRS) test was used to compare the differences in TMB, stromal score, immune score, and tumor purity between male and female patients for both pan‐cancer and tumor type‐specific analysis. Moreover, TMB has been proposed to be correlated with TME‐related features (immune scores, stromal scores, tumor purity, and immune cells) in several cancer types [20, 21]. Investigation of differential correlations of TMB with TME‐related features between male and female patients may reveal new insights into the sex differences in TME. To do this, we first tested the correlation between TMB and tumor microenvironment features (including stromal score, immune score, tumor purity, and immune cells) using Spearman correlate analysis. The differential correlation analysis was then performed by comparing the correlations of TMB with a tumor microenvironment feature (stromal score, immune score, tumor purity, or immune cells) in male patients and female patients. To test whether the correlation coefficients in males and females were significantly different, we used Fisher’s transformation method to transform correlation coefficients for each of male and female group, *r*_male_ and *r*_female_, into *Z*_male_ and *Z*_female_, respectively, using the following formula:

(1) $$Z_{\mathrm{male}}=\frac{1}{2}\ln\frac{1+r_{\mathrm{male}}}{1‐r_{\mathrm{male}}}$$

where *r* is the Spearman correlation coefficient between TMB and a microenvironment feature in male groups. Similarly, we transformed the coefficient *r*_female_ into *Z*_female_. Differences between the two correlations can be tested using the following formula:

(2) $$Z‐\text{score}=\frac{Z_{\text{male}}‐Z_{\text{female}}}{\sqrt{\frac{1}{n_{1}‐3}+\frac{1}{n_{2}‐3}}}$$

where *n*_1_ and *n*_2_ are the numbers of male and female patients. The *Z*‐value has an approximately Gaussian distribution [30] and the *P*‐value can be obtained with standard normal distribution according to the *Z‐*score.

The gene expression values and immune cell infiltration levels were compared in the male and female patients using an unpaired two‐sided *t*‐test for each cancer type, and the *P*‐values were adjusted using the false discovery rate (FDR) method proposed by Benjamin and Hochberg [31].

### 2.5 Identification of sex differences in immune function pathways

The immune function pathways were downloaded from the ImmPort database (https://www.immport.org/shared/home). The GSEA method was applied to identify the immune function pathways of sex differences for each cancer type. Specifically, we compared the gene expression values between female and male patients with the *t*‐test, and the gene sets of immune pathways were respectively assigned to a ranked gene list based on the T‐score of expression values. If the genes in a pathway were enriched at the top or bottom of the list, the pathway will tend to be female‐biased or male‐biased. For each pathway, the normalized pathway enrichment score, its corresponding *P*‐value, and FDR were calculated through a permutation test in a cancer type.

### 2.6 Identification of the immune cells driven by sex‐biased mutation genes

We first identified the sex‐biased mutation genes. For a cancer type, we focused on non‐silent somatic mutations (nonsense, missense, splice‐site mutations, stop codon read‐throughs, change of start codon, frame‐shift indels, and inframe indels) in the protein‐coding region of genes. A binary gene mutation matrix was constructed according to the MAF file, whose element is 1 (true) if any mutation occurs in a particular gene in a particular sample; otherwise it is 0 (false). To provide potential biological significance and detecting power in our analysis, the genes with more than 5% mutation frequency were retained in each cancer type. Pearson’s Chi‐square test (continuity correction was used if needed) was used to compare gene mutation versus non‐mutation status between male and female patients. The *P*‐values were then adjusted with the FDR method [31]; FDR < 0.25 was considered significant.

We then identified the immune cell responses triggered by sex‐biased mutation genes. The multivariate logistic regression analysis was performed to estimate the effect of sex‐biased mutation genes on immune cells in the individual cancer type.

### 2.7 Prognostic models of sex‐specific immune cells

As the immune cells for male and female patients of cancer may be different, inference of sex‐specific prognostic signature of immune cells is important. Associations between the infiltration level of each immune cell type and survival were tested using a univariate Cox proportional hazard regression model in male and female patients, respectively. With Cox *P*‐value < 0.05, the male‐ and female‐specific prognostic cells were obtained in male and female patients, respectively. A male‐ or female‐specific risk score for every patient in male or female groups was calculated using a prognostic score model based on the infiltration levels of the immune cells. The prognostic score model was as follows:

(3) $$Riskscore=\sum_{k\in s}\beta_{k}\alpha_{k}$$

where *S* is a set of male‐ or female‐specific prognostic cells; *a_k_* is the infiltration level of cell *k;* β*_k_* is the regression coefficient of the univariate Cox proportional hazard regression model estimated on *a_k_* and the overall survival data in male or female patient groups. A high‐risk score indicates poor survival for patients. According to the median of male‐ or female‐specific risk scores, male and female patients were classified into high‐risk and low‐risk groups. Kaplan–Meier curves for male‐ or female‐specific survival were generated in male and female patient groups, and were compared using the log‐rank test.

## 3 Results

**Fig. 1:**
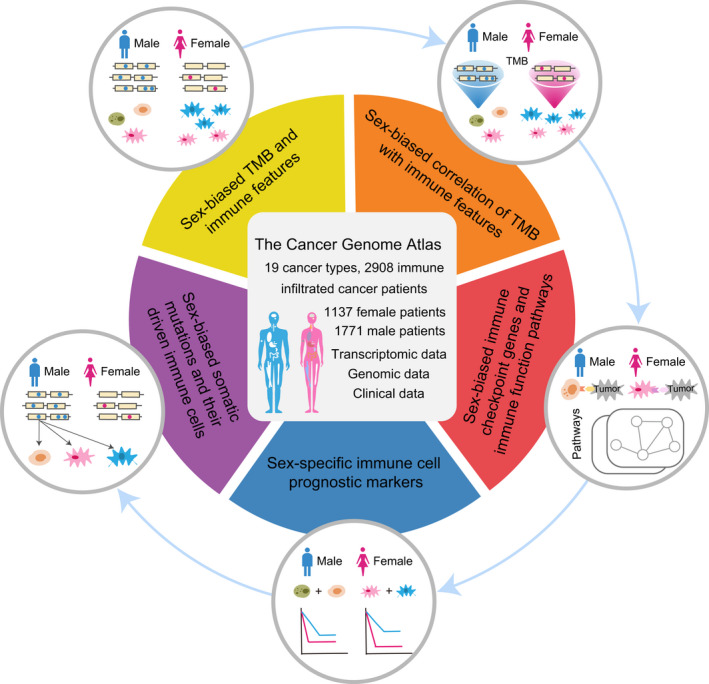
Overview of sex‐based differences analysis of cancer immune‐related features.

We focused on primary cancer samples with at least moderate immune infiltration. The CIBERSORT algorithm was used to estimate the immune infiltration extent of cancer (see Materials and methods). With CIBERSORT *P* < 0.05, we obtained 19 cancer types from TCGA with sufficient male and female sample sizes (≥ 10 for both); the cancer types include bladder urothelial carcinoma (BLCA), colon adenocarcinoma (COAD), diffuse large B‐cell lymphoma (DLBC), glioblastoma multiforme (GBM), head and neck squamous cell carcinoma (HNSC), kidney renal clear cell carcinoma (KIRC), kidney renal papillary cell carcinoma (KIRP), acute myeloid leukemia (LAML), brain lower grade glioma (LGG), liver hepatocellular carcinoma (LIHC), lung adenocarcinoma (LUAD), lung squamous cell carcinoma (LUSC), pancreatic adenocarcinoma (PAAD), rectum adenocarcinoma (READ), sarcoma (SARC), skin cutaneous melanoma (SKCM), stomach adenocarcinoma (STAD), thyroid carcinoma (THCA), and thymoma (THYM). There are 2908 cancer samples in total (1771 male and 1137 female samples) (Table S1), and the corresponding molecular data and clinical phenotype data were downloaded. A comprehensive analysis was performed to investigate sex‐based differences of TMB, TME cells, immune scores, immune‐associated genes, and functional pathways, etc., in pan‐cancer and each individual cancer type (Fig. 1).

### 3.1 Sex‐biased TMB and immune features

**Fig. 2:**
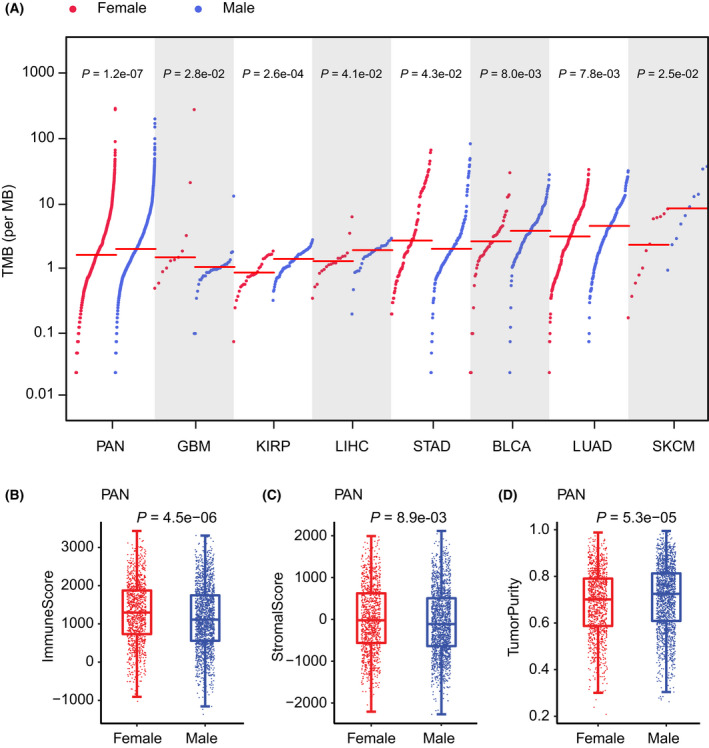
Sex differences in TMB, immune scores, stromal scores, and tumor purity. (A) Prevalence of TMB of male and female patients in pan‐cancer (1771 male and 1137 female patients), BLCA (119 male and 51 female patients), KIRP (83 male and 29 female patients), LIHC (29 male and 18 female patients), LUAD (190 male and 228 female patients), and SKCM (10 male and 12 female patients). Red lines show median TMB for each group. (B) Box plots of immune scores of 1771 male and 1137 female patients in pan‐cancer. (C) Box plots of stromal scores of 1771 male and 1137 female patients in pan‐cancer. (D) Box plots of tumor purity of 1771 male and 1137 female patients in pan‐cancer. The statistical significance (*P*‐value) for each feature was determined by the Wilcoxon rank‐sum test. The top and bottom error bars indicate the 90th and 10th percentiles of each score.

We first tested the difference in pan‐cancer TMB between male and female patient groups. TMB of male patients was significantly higher than that of female patients (WRS test *P* = 1.2e‐07). As tumor characteristics in the patients in different cancer types are frequently different, we then tested whether there were sex differences within individual cancer types. Seven of these cancers showed significant sex differences (Fig. S2); BLCA, KIRP, LIHC, LUAD, and SKCM were male‐biased (WRS test *P* = 0.008, 0.00026, 0.041, 0.0078, and 0.025 respectively); GBM and STAD were female‐biased (WRS test *P* = 0.028 and 0.043). The prevalence of TMB of male and female patients across pan‐cancer and these seven cancers is shown in Fig. 2A.

To validate the sex‐biased TMB, we collected somatic mutation data from the cBioPortal and ICGC data platform (the data sources were listed in Table S3) for the cancers which showed significant sex‐biased TMB in TCGA. We obtained almost consistent results in the validation sets, that is, male patients showed higher TMB than female patients in BLCA, LUAD, LINC, SKCM, and renal cell carcinoma (URCC), and the female patients showed higher TMB than male patients in GBM (Fig. S3).

We further tested the sex difference of immune scores, stromal scores, and tumor purity in pan‐cancer. We found that the immune scores and stromal scores were female‐biased (WRS test *P* = 4.5e‐06 and 0.0089) and tumor purity was male‐biased (WRS test *P* = 5.3e‐05) (Fig. 2B‐D). We also analyzed whether there were sex differences of these features within individual cancer types and focused our analysis on each cancer type (Fig. S4–S6). Specifically, the immune scores of female patients in HNSC, LUAD, and LUSC (WRS test *P* = 0.018, 0.018, and 0.0019, respectively) are significantly higher than those in male patients, but lower than those in male patients in KIRP and SARC (WRS test *P* = 0.041 and 0.044) (Fig. S4). For the stromal scores, the female patients showed higher LUAD compared with the male patients (WRS test *P* = 0.048) (Fig. S5), which is lower than the male patients in SARC (WRS test *P* = 0.022). Additionally, the tumor purity in SARC was significantly higher in female patients than male patients (WRS test *P* = 0.024), but lower than male patients in HNSC, LUAD, and LUSC (WRS test *P* = 0.038, 0.018, and 0.034, respectively). The female patients in KIRP also exhibited higher tumor purity than male patients, with statistical significance just exceeding the threshold (WRS test *P* = 0.061) (Fig. S6). Taken together, the male patients generally have higher TMB and tumor purity, but lower immune scores and stromal scores compared with female patients in pan‐cancer. However, there are some contrary results in some individual cancer types. For example, the female patients were shown to have higher immune scores and stromal scores, but lower tumor purity compared with male patients in LUAD. In contrast, the female patients had lower immune scores and stromal scores, but higher tumor purity compared with male patients in SARC. Our results suggest a divergent sex difference of immune features across different cancer types.

**Fig. 3:**
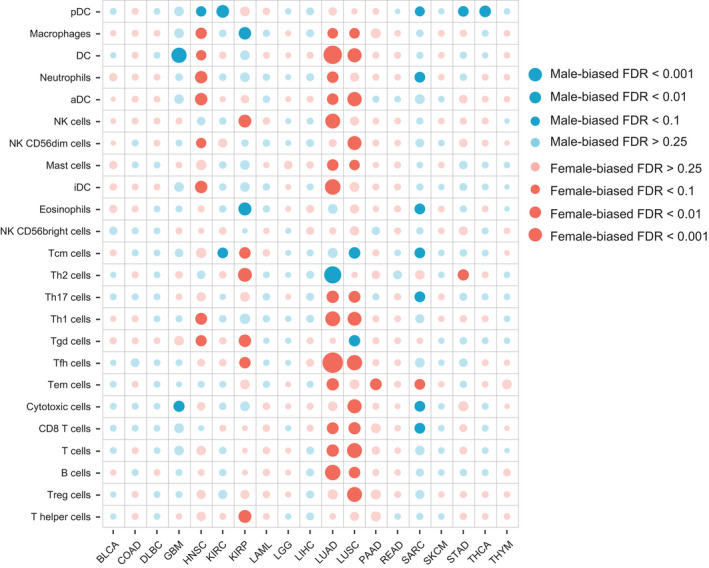
Bubble plot of sex‐biased immune cells. Differences of the relative infiltration levels of 24 immune cell populations between male and female patients across 19 cancer types: BLCA (119 male and 51 female patients), COAD (91 male and 71 female patients), DLBC (16 male and 20 female patients), GMB (36 male and 11 female patients), HNSC (297 male and 111 female patients), KIRC (154 male and 76 female patients), KIRP (83 male and 29 female patients), LAML (27 male and 28 female patients), LGG (55 male and 40 female patients), LIHC (29 male and 18 female patients), LUAD (190 male and 228 female patients), LUSC (309 male and 110 female patients), PAAD (46 male and 40 female patients), READ (21 male and 17 female patients), SARC (68 male and 71 female patients), SKCM (10 male and 12 female patients), STAD (144 male and 83 female patients), THCA (29 male and 77 female patients), THYM (47 male and 44 female patients). The statistical significance (*P*‐value) for each cell was determined by the *t*‐test, and which was then adjusted by FDR. The immune cells with FDR < 0.25 are shown with prominent bubbles.

Finally, we tested the sex‐biased immune cells across different cancer types. We collected 24 immune cell type‐specific gene signatures from the Bindea et al. publication [28] (Table S2). The ssGSEA algorithm was applied to the normalized gene expression data to estimate the relative infiltration level for each cell. To identify sex‐biased immune cell infiltration patterns, we compared the cell infiltration levels between female and male patients in each cancer type using the *t*‐test. The detailed results of sex‐biased immune cells for each cancer type are listed in Table S4. With FDR < 0.25, 10 of 19 cancers have at least one sex‐biased immune cell. In general, we observed several cancer types to have female‐biased immune cells, including HNAC, KIRP, LUAD, and LUSC. For example, 14 of 24 immune cells were shown to have higher relative infiltration levels in the female patients of LUAD (Fig. 3). The two most significant female‐biased cells in the cancer are follicular helper T (Tfh) cells and dendritic cells (DC; *t*‐test FDR = 2.24e‐06 and 5.72e‐05); the Tfh cells were demonstrated to be involved in the antitumor immunity and were associated with better clinical outcomes in non‐small cell lung cancer (NSCLC) [32] and the DC were proposed to be the key factors providing protective immunity against lung cancers [33]. These results suggest that the Tfh cell and DC activities for male patients in LUAD should be enhanced. In contrast, several cancer types (GBM, KIRC, and SARC, etc.) had male‐biased immune cells (Fig. 3). For example, seven immune cells, including plasmacytoid dendritic cells (pDC), neutrophils, eosinophils, T central memory (Tcm) cells, T helper 17 (Th17) cells, cytotoxic cells, and CD8 T cells, were shown to have higher infiltration levels in male SARC patients. Moreover, pDC, which have been shown to contribute to cancer pathogenesis [34] were also observed to be male‐biased in HNSC, KRIC, STAD, and THCA. These results suggest that in male patients of these cancers, pDC should be inactivated.

### 3.2 Sex‐biased correlation of TMB with immune features

**Fig. 4:**
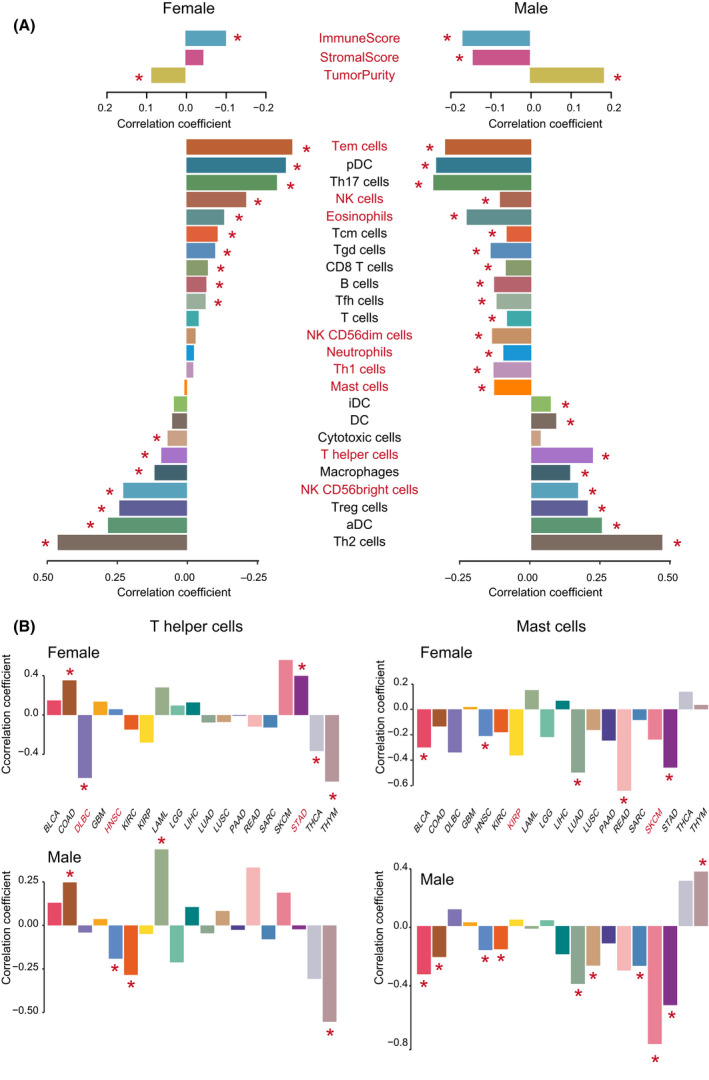
Sex differential correlation of TMB with immune features. (A) Correlation of TMB with immune features (immune score, stromal score, tumor purity, and immune cell infiltration levels) in 1771 male and 1137 female patients in pan‐cancer. Spearman correlation coefficients are computed, and are plotted on the x‐axis in bar plots. Red asterisks indicate that the level of significance of correlation coefficients was < 0.05 in male or female patient groups. Immune features with red labels indicate that the correlations of TMB with the features are significant differences between 1771 male and 1137 female patients in pan‐cancer (Fisher’s transformation method, *P* < 0.05). (B) Correlation of TMB with infiltration levels of Th cells and mast cells in male and female patients across 19 cancer types: BLCA (119 male and 51 female patients), COAD (91 male and 71 female patients), DLBC (16 male and 20 female patients), GMB (36 male and 11 female patients), HNSC (297 male and 111 female patients), KIRC (154 male and 76 female patients), KIRP (83 male and 29 female patients), LAML (27 male and 28 female patients), LGG (55 male and 40 female patients), LIHC (29 male and 18 female patients), LUAD (190 male and 228 female patients), LUSC (309 male and 110 female patients), PAAD (46 male and 40 female patients), READ (21 male and 17 female patients), SARC (68 male and 71 female patients), SKCM (10 male and 12 female patients), STAD (144 male and 83 female patients), THCA (29 male and 77 female patients), THYM (47 male and 44 female patients). Spearman correlation coefficients are computed, and are plotted on the y‐axis in bar plots. Red asterisks indicate that the level of significance of correlation coefficients was less than 0.05 in male or female patient groups. Cancer types with red labels indicate that the correlations of TMB with the features are significant differences between male and female patient groups (Fisher’s transformation method, *P* < 0.05).

As TMB was proposed to potentially correlated with cancer immune infiltrates in previous studies, we tested the differential correlation of TMB with TME features (immune score, stromal score, tumor purity, and immune cell infiltration level) between male and female cancer patients (Table S5). We found that the TMB was negatively correlated with immune score and stromal score, and positively correlated with tumor purity in both male and female pan‐cancer patients. Interestingly, these correlations all showed significant sex differences (Fisher’s transformation method, *P* = 0.038, 0.005, and 0.004, respectively), male patients possessing greater correlation than female patients (Fig. 4A). We then found nine immune cells to have a sex differential correlation with TMB in pan‐cancer patients (*P* < 0.05, Table S5), including T effector memory (Tem) cell, NK cell, eosinophils, NK CD56dim cell, neutrophils, T helper 1 (Th1) cell, mast cell, T helper (Th) cell and NK CD56bright cell (these cells names are marked with red in Fig. 4A).

The most significant is mast cell (Fisher’s transformation method, *P* = 9.1e‐05), which showed a significant negative correlation with TMB in male patients (marked with a red star) but did not show a correlation in female patients. Mast cells are proposed to play important roles in the control of innate and adaptive immunity, endowing them with the ability to tune the nature of host responses to cancer and ultimately influence the outcome and fate of the cancer patient [35].

The second significant cell is the Th cell (Fisher’s transformation method, *P* = 3.9e‐04), which showed a greater positive correlation with TMB in male than in female patients. Knutson et al. reported that Th cells were central to the development of an immune response by activating antigen‐specific effector cells [36]. These results suggest that it should consider sex differential correlation of some immune features with TMB in the development of immunotherapeutic drugs and methods.

We then tested the sex differential correlation of TMB with immune cell infiltration level across different cancer types (Table S6). For the Th cells, three cancers, DLBC, HNSC, and STAD (Fisher’s transformation method, *P* = 0.026, 0.012, and 7.7e‐04), were found to have a sex‐biased correlation with TMB (the cancer names are marked in red in Fig. 4B). Specifically, Th cells showed a significant negative correlation with TMB in female patients of DLBC (Spearman correlation coefficients *r *= −0.64, *P* = 0.0024) but did not show a significant correlation in male patients of DLBC. Moreover, Th cells were found to have a positive correlation with TMB in female patients of STAD (Spearman correlation coefficients *r* = 0.40, *P* = 1.9e‐04); however, the cells did not found to be significantly correlated with TMB in the male patients of STAD. We also observed that Th cells had a significant negative correlation with TMB only in male patients of HNSC (Spearman correlation coefficients *r *= −0.19, *P* = 9.2e‐04). For mast cells, a sex‐biased correlation was found with TMB in KIRP and SKCM (Fisher’s transformation method, *P* = 0.03 and 0.04) (Fig. 4B). The mast cells of female patients of KIRP were shown to be negatively correlated with TMB (Spearman correlation coefficients *r *= −0.36, *P* = 0.053), but the cells of male patients of KIRP did not show a significant correlation with TMB (Spearman correlation coefficients *r* = 0.04, *P* = 0.69). In SKCM, the mast cells of male patients showed a significantly greater negative correlation with TMB (Spearman correlation coefficients *r *= −0.81, *P* = 0.0082) compared with female patients. Our results provide insights into the sex differential correlation between TMB and tumor immune cells in different types of cancers.

### 3.3 Sex‐biased immune checkpoint genes and immune function pathways

**Fig. 5:**
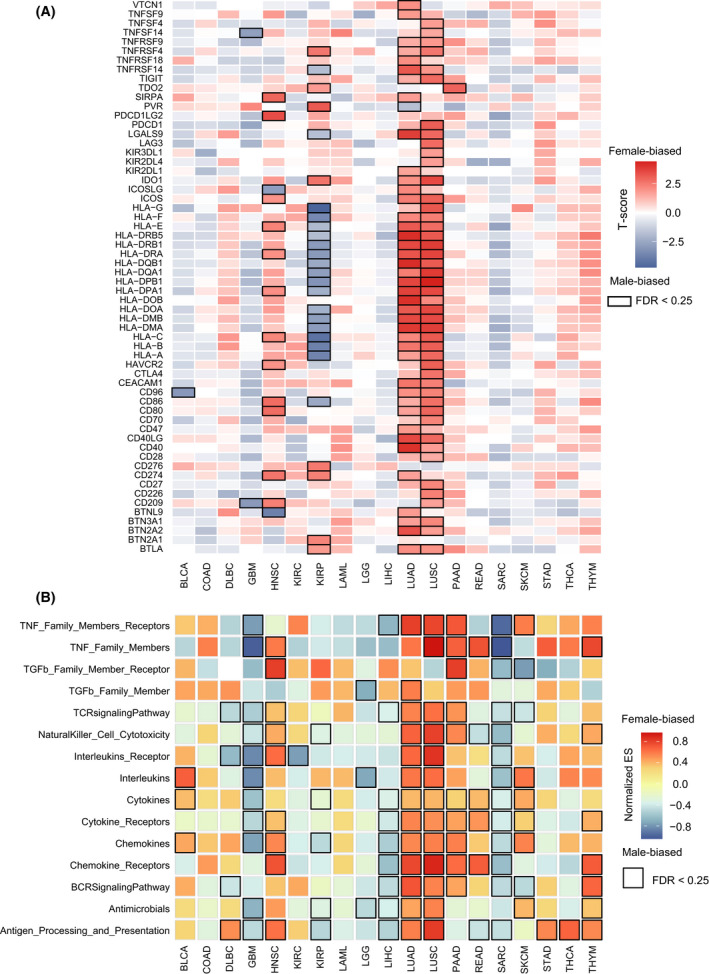
Sex‐biased immune checkpoint genes and immune function pathways across 19 cancer types: BLCA (119 male and 51 female patients), COAD (91 male and 71 female patients), DLBC (16 male and 20 female patients), GMB (36 male and 11 female patients), HNSC (297 male and 111 female patients), KIRC (154 male and 76 female patients), KIRP (83 male and 29 female patients), LAML (27 male and 28 female patients), LGG (55 male and 40 female patients), LIHC (29 male and 18 female patients), LUAD (190 male and 228 female patients), LUSC (309 male and 110 female patients), PAAD (46 male and 40 female patients), READ (21 male and 17 female patients), SARC (68 male and 71 female patients), SKCM (10 male and 12 female patients), STAD (144 male and 83 female patients), THCA (29 male and 77 female patients), THYM (47 male and 44 female patients). (A) Heatmaps of immune checkpoint genes for each cancer type. Differential expression extents (*t*‐scores) of genes between male and female patients are calculated by *t*‐test, and female‐biased and male‐biased genes are shown in red and blue, respectively. Boxes highlight the statistically significant immune checkpoint genes (FDR < 0.25). (B) Heatmaps of immune function pathways for each cancer type. Sex‐biased immune function pathways are identified by GSEA based on the sex‐biased gene ranks of mRNA expression for each cancer type, and normalized enrichment score (ES) for each pathway is also calculated by GSEA. Female‐biased and male‐biased pathways are shown in red and blue, respectively. Boxes highlight the statistically significant enriched pathways (FDR < 0.25).

To test whether the immune checkpoint genes (ICG) differ between male and female cancer patients, we collected 68 ICG from the Hu et al. study [37]. The unpaired two‐sided *t*‐test was used to compare the gene expression values between the female and male patients for each cancer type. The statistically significant *P*‐values were adjusted using the false discovery rate (FDR) method. It was shown that 88% of genes (60/68) were sex‐biased, including some well‐known ICG such as PDCD1, CD274, and CTLA4. Moreover, we found that seven of 19 cancers have at least one ICG with a sex difference with FDR < 0.25: BLCA, GBM, HNSC, KIRP, LUAD, LUSC, and PAAD (Fig. 5A). The detailed information on ICG for each cancer type is listed in Table S7. LUAD, LUSC, and HNSC tend to have more female‐biased ICG (Female > Male), whereas KIRP and GBM tend to have more male‐biased ICG (Male > Female). Specifically, there are 31 female‐biased ICG in LUAD; these genes include some genes of the human leukocyte antigen (HLA) family (e.g. HLA‐A, HLA‐B, HLA‐C, HLA‐DMA, and HLA‐DPB1), CD274, CD40, CD80, CD86, some genes of TNF receptor superfamily (e.g. TNFRSF4, TNFRSF9, TNFRSF18). In contrast, some genes of the HLA family (e.g. HLA‐A, HLA‐B, HLA‐C, HLA‐DMA, and HLA‐DPB1, etc.) were shown to be male‐biased in KIRP. Moreover, several ICG (e.g. CD274, CD276, and TNFRSF4) were found to be female‐biased in KIRP (Fig. 5A). It has been reported that the absence of HLA‐I (e.g. HLA‐A, HLA‐B, HLA‐C) expression is a common finding in different tumor tissues and is a major mechanism used by tumor cells to escape T cell‐mediated immune surveillance [38]. Studies have also shown that the expression of PD‐L1 protein (encoded by the CD274 gene) in tumor cells has been proposed as a predictive biomarker of response to anti‐PD‐1/PD‐L1 treatment [39, 40, 41]. Our results suggest that sex differences of ICG in the specific cancer types (e.g. KIRP, LUAD, and LUSC) should be considered for the immune checkpoint blockade therapies.

We then test the sex‐biased immune function pathways across different cancer types. For each cancer, we compared the gene expression values between female and male patients with the *t*‐test, and a gene ranked list was constructed according to the *t*‐scores of genes. The immune function pathways downloaded from the ImmPort database were mapped on the ranked gene list, and the GSEA method was applied to identify sex‐biased pathways. With FDR < 0.25, 16 of 19 cancers have at least one sex difference pathway (Fig. 5B). The detailed information on pathways for each cancer type are listed in Table S8. Interestingly, GBM, KIRP, LGG, LIHC, and SARC tended to have more male‐biased pathways, and HNSC, LUAD, LUSC, PAAD, and THYM more female‐biased pathways. For the significant immune pathways, the TNF Family Members, receptors, cytokines, cytokine receptors, and chemokines showed a consistently high expression for male patients in GBM, LIHC, and SARC, and for female patients in LUAD, LUSC, and PAAD.

**Fig. 6:**
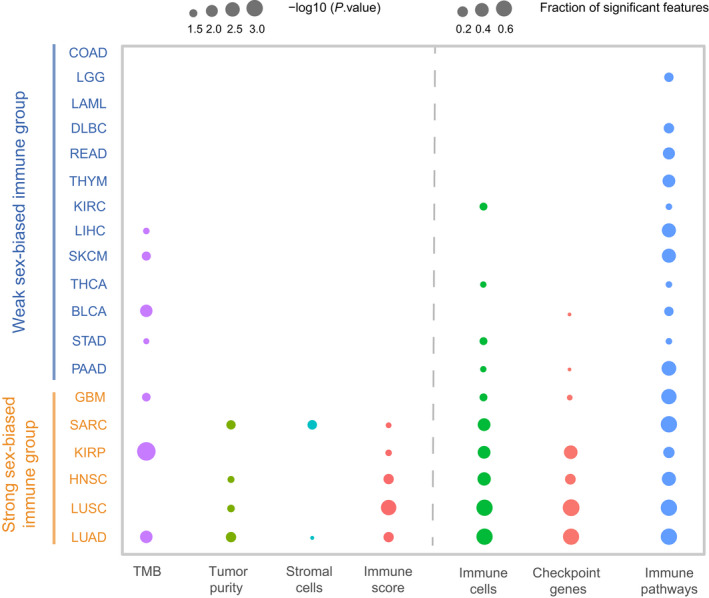
Classification of cancer types. The cancer types are classified into ‘strong sex‐biased’ and ‘weak sex‐biased’ immune groups. The statistically significant level (*P*‐value < 0.05) for TMB, immune scores, stromal scores, and tumor purity as well as the fraction of significant features (FDR < 0.25) for immune cells, immune checkpoint genes, and functional pathways were shown for each of cancer types. The ‘strong sex‐biased’ immune group and ‘weak sex‐biased’ immune group are marked in orange and blue, respectively. The statistical significance (*P*‐value) for TMB, immune scores, stromal scores, or tumor purity was determined by the Wilcoxon rank‐sum test.

Overall, according to the sex differences of immune‐related features, the cancer types could be classified into a ‘‘strong sex‐biased’ immune group and a ‘weak sex‐biased’ immune group. For the statistically significant level (*P*‐value) of TMB, immune scores, stromal scores, tumor purity, and immune cells as well as the fraction of significant features for immune cells, immune checkpoint genes, and functional pathways, the cancer types with more than half (at least four of seven features) of significant sex‐biased features were labeled as the ‘strong sex‐biased’ immune group, and the others as the ‘weak sex‐biased’ immune group (Fig. 6). The strong sex‐biased immune group includes LUAD, LUSC, HNSC, KIRP, SARC, and GBM, which shows much more extensive sex‐biased TME signatures; we should pay attention to the significant sex‐biased feature for these cancer types in the immunotherapy of cancer.

### 3.4 Sex‐specific immune cell prognostic markers

The immune cells have been proposed to be associated with the prognosis of cancer patients in our previous study, and we wanted to see whether there were any differences between male and female cancer patient cohorts. For each cancer type, a univariate Cox proportional hazards model was used to estimate the hazard ratio (HR) of overall survival and statistical significance level (*P*‐value) for each immune cell in males and females, respectively (Table S9). Cross cancer assessment of prognostic analysis in male and female patients revealed significant immune cells (Wald *P* < 0.05) (Fig. S7). Seven recurrently prognostic immune cells (at least four cancer types) were identified in male patient cohorts, including Th2 cell, T cell, DC, and NK cells. Th2 cell was proposed to promote tumor progression through the secretion of cytokines interleukin (IL)‐4, IL‐6, IL‐10, and IL‐13 to activate tumor‐associated M2 macrophages [42], and which was found to be a risk factor of male patients in SARC, LUAD, KIRP, PAAD, KIRC, and LIHC (HR > 1, Wald *P* < 0.05). We also found that the T cell played a large role in our fight against cancer as a protective factor in male patients in BLCA, HNSC, LUAD, and PAAD (HR < 1, Wald *P* < 0.05). Additionally, four recurrently prognostic immune cells (at least four cancer types) were identified in female patient cohorts: Th17 cells, Tem cells, NK cells, and CD8 T cells (Fig. S7). For example, Th17 cell was found to be a protective factor in female patients in LUAD, COAD, STAD, and LAML. Tem cell was found to be a protective factor in female patients in LUAD and PAAD, but a risk factor in female patients in STAD and LUSC. The recurrently prognostic immune cells were observed in male or female patients across different cancer types, highlighting the repurposing potential of immunotherapy drugs targeting these cells.

**Fig. 7:**
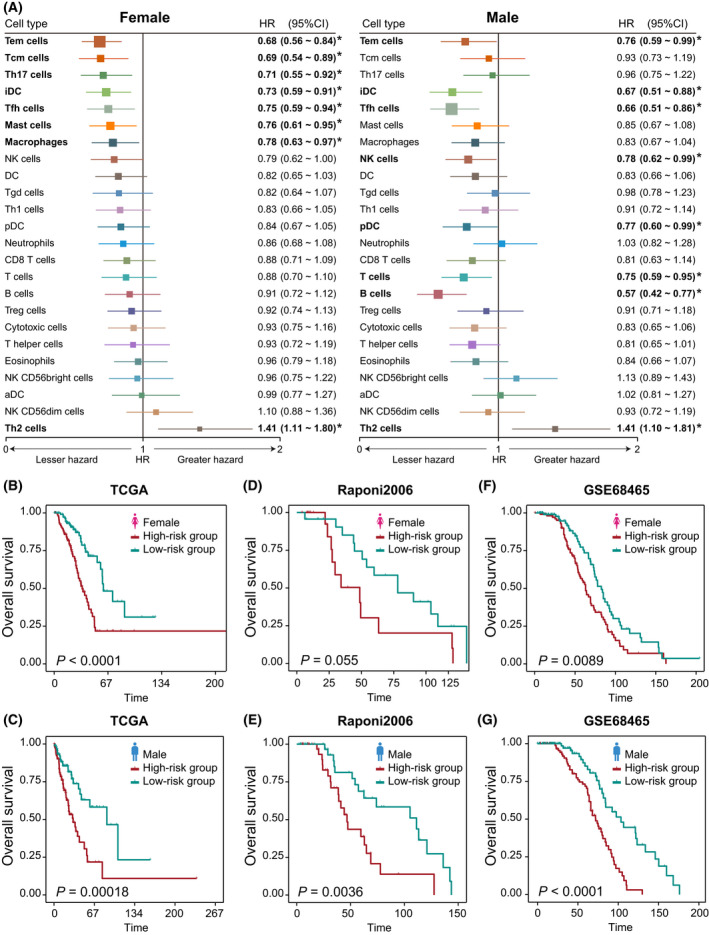
Prognostic associations of immune cells in LUAD. (A) Forest plots of the univariate Cox hazard model for overall survival in 190 male and 228 female patients, respectively. HR (boxes) and 95% confidence intervals (horizontal lines) are shown. Box size is inversely proportional to the width of the confidence interval. Asterisks denote estimates with a *P*‐value < 0.05. Kaplan–Meier survival curves of patients classified into high‐ and low‐risk groups: (B) 228 female patients of TCGA‐LUAD using the female prognostic score model; (C) 190 male patients of TCGA‐LUAD using the male prognostic score model; (D) 46 female patients of Raponi et al. set using the female prognostic score model; (E) 81 male patients of Raponi et al. dataset using the male prognostic score model; (F) 220 female patients of GSE68465 datasets using the female prognostic score model; (G) 223 male patients of GSE68465 datasets using the male prognostic score model. *P*‐values are calculated by log‐rank test. Vertical hash marks indicate censored data.

As LUAD shows much more extensive sex‐biased immune‐related features (TMB, immune scores, stromal scores, tumor purity, immune cells, immune cells, immune checkpoint genes, and functional pathways) compared with other cancer types (Fig. 6), we took LUAD as an example to investigate further whether the sex‐specific signatures based on sex‐specific cells could classify the male and female patients into high‐risk and low‐risk groups, respectively. With Wald *P* < 0.05 in univariate Cox proportional hazards model, we identified eight significant immune cells for female patients (Fig. 7A): Tem cell, Tcm cell, interstitial dendritic cell (iDC), Th17 cell, Tfh cell, mast cell, and macrophages are protective factors (HR < 1) and Th2 cell, a risk factor (HR > 1). In contrast, there were eight significant immune cells for male patients: Tem cell, iDC, pDC, Tfh cell, NK cell, T cell, and B cell are protective factors, and Th2 cell, a risk factor. Although four significant cells overlap between male and female patients, their association with the extent of survival differs between these patients. With the sex‐specific cells, the male‐ and female‐specific prognostic signatures were constructed. For these signatures, we respectively calculated the risk score of signature for every male and female patient based on the cell infiltration level; the risk score formulas are listed in the Supporting Information. We then used the Kaplan–Meier method and log‐rank test to evaluate the power of classification of risk scores in male and female patients. According to the median of male‐ and female‐risk scores, male and female patients were classified into high‐risk and low‐risk groups (log‐rank test *P* < 1.0e‐4 for female patients; *P* = 1.8e‐4 for male patients) (Fig. 7B,C). We further performed multivariate Cox proportional hazard regression analysis in male and female LUAD patient groups to test whether the male‐ and female‐risk scores are independent prognostic factors compared with other clinical characters, including age and clinical stage. The results showed that both male‐ and female‐risk scores could serve as independent prognostic factors for OS after multivariable adjustment by clinical characters in male and female LUAD patient groups, respectively (Table S10).

To determine whether the male‐ and female‐specific cells had the same or similar prognostic value in independent datasets, we then applied the same method to classify male and female patients from two independent lung adenocarcinoma datasets, Raponi et al. [43] and GSE68465 [44]. For each independent validation set, male and female patients were respectively divided into high‐risk and low‐risk groups (log‐rank test, *P* = 0.055 and *P* = 0.0036 for female and male patients in Raponi et al. dataset, Fig. 7D and E; *P* = 0.0089 and *P* < 1.0e‐4 for female and male patients in GSE68465, Fig. 7F and G). Patients with high‐risk scores had significantly shorter overall survival than those with low‐risk scores.

Furthermore, several other cancer types (such as LUSC and HNSC) also showed sex‐biased features, except LUAD (Fig. 6). Therefore, we also constructed the sex‐specific risk score models in the strong sex‐biased cancer types. The detailed results are listed in the Supporting Information and Fig. S8.

### 3.5 Sex‐biased somatic mutations and their driven immune cells

**Fig. 8:**
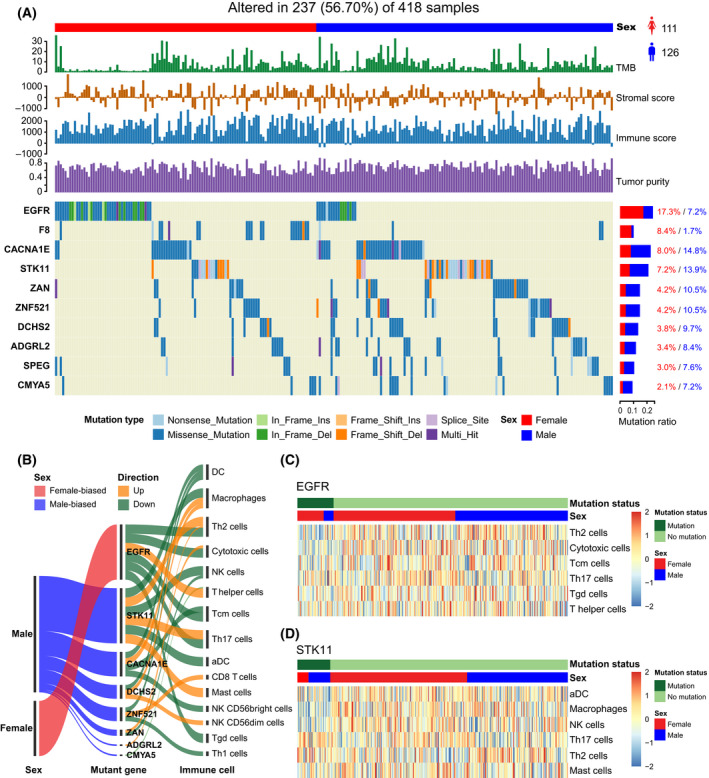
Sex‐biased somatic mutations and their driven immune cells in LUAD. (A) Waterfall plot of sex‐biased somatic mutation genes in 237 LUAD samples (126 male and 111 female patients). The statistical significance (*P*‐value) was determined by the Chi‐square test, which was then adjusted by FDR. Genes with Chi‐square test FDR < 0.25 are shown. Samples are displayed as columns with the sex label on the top, and TMB, immune scores, stromal scores, tumor purity, and different types of somatic mutations are shown below. The bar plots next to the waterfall plot show the mutation frequencies of male and female patients. (B) Sankey diagram demonstrates the immune cells associated with sex‐biased somatic mutation genes based on 418 LUAD patients (190 male and 228 female patients). (C) Heatmaps of the infiltration levels of immune cells between patients with mutation of EGFR in based on 418 LUAD patients. (D) Heatmaps of the infiltration levels of immune cells between patients with mutation and no mutation of STK11 based on 418 LUAD patients.

To identify sex‐biased somatic mutations, the genes with high frequency mutations were retained in each cancer type (> 5% mutated samples). We focused on LUAD, which showed significant sex differences in various immune features (TMB, immune score, stromal score, and tumor purity, etc.). Pearson’s Chi‐square test was used to identify sex‐biased somatic mutations (See Materials and methods). At FDR = 0.25 in the Chi‐square test, we identified two female‐biased and eight male‐biased somatic mutation genes in LUAD (Fig. 8A). The detailed results are listed in Table S11. EGFR mutations were identified to be female‐biased (male versus female: 7.2% versus 17.3%, Chi‐square test FDR = 0.20) in LUAD patients. Interestingly, the mutations were proposed to be associated with tyrosine kinase inhibitor (gefitinib) response and prolonged survival in NSCLC patients [45], which were also proposed to result in a decline of immune infiltration or a lack of infiltrating immune cells in the NSCLC microenvironment [46]. STK11was identified to be male‐biased (male versus female: 14.8% versus 8%, Chi‐square test FDR=0.15), which was consistent with previous studies [47, 48]. However, we found this gene to be more frequently mutated in males than in females for the immune‐infiltrated LUAD cohort. Moreover, Koyama et al. proposed that STK11/LKB1 deficiency may promote neutrophil recruitment and proinflammatory cytokine production to suppress T‐cell activity in the lung tumor microenvironment [49].

Further to estimate which immune cell responses may be potentially triggered by sex‐biased mutation genes, we performed the multivariate logistic regression analysis on gene mutation status and cell infiltration levels in LUAD (Fig. 8B); eight of 10 sex‐biased mutation genes were associated with at least one immune cell. The statistically significant cells associated with these mutations are listed in Table S12. For example, we found that EGFR mutations were associated with six immune cells (multivariate logistic regression *P* < 0.05), five cells of which are downregulated (Th2 cells, cytotoxic cells, Tcm cells, Th17 cells, T gamma delta (Tgd) cells), and one cell upregulated (T helper cell) in EGFR mutation patients (Fig. 8C). For the patients with STK11 mutations, three cells were downregulated (aDC, macrophages, and NK cells) and three cells upregulated (Th17 cells, Th2 cells, and Mast cells) (Fig. 8D). These results may help understanding of potential immune cells driven by sex‐specific mutations in order to implement precise treatment.

### 3.6 Sex differences of TMB’s ability in predicting the response to immunotherapy

TMB was proposed to be a predictive biomarker of the immune checkpoint blockade therapy response in previous studies. We wanted to know whether the TMB predictive power showed sex differences. Three public ICB cancer therapy datasets (anti‐PD‐1, anti‐PD‐L1, or anti‐CTLA‐4), including pan‐cancer [50], non‐small cell lung cancer [51], and clear cell renal cell carcinoma [52], were collected from the cBioPortal website (http://www.cbioportal.org/). TMB was used to predict the response to ICB therapies in male and female patients, respectively, for each dataset. The receiver operating characteristic curve analysis was performed, and the area under the curve (AUC) was used to evaluate the predictive power of TMB. The results showed that the AUC in female patients is larger than in male patients for each dataset (Fig. S9), which indicates that TMB has a better predictive power for females than for males in cancer. These results are consistent with a previous study [53], which tested sex differences in the performance of TMB in ICB response prediction only in lung cancer. The female‐favored predictive power of the TMB may be due to the female patients having a stronger immune score (immune microenvironment) compared with male patients (Fig. 2B). The female patients with high TMB are highly immunogenic and may exhibit stronger immunosuppressive signals. ICB therapy may block these signals between tumor cells and immune cells, and thus the tumor can be attacked more effectively in female patients than in male patients.

## 4 Discussion

Sex is a key factor that has been proposed to be associated with cancer initiation, progression, and prognosis outcome in multiple cancer types [1, 2]. Although the significance of the sex effect in cancer immune responses is known from some of the literature [5, 8], its immune microenvironment characterization has largely remained elusive. A comprehensive analysis of immune‐related feature differences between male and female cancer patients is urgently needed. Our study focuses on sex‐based differences of TMB, TME features (immune scores, stromal scores, tumor purity, and immune cells), immune checkpoint‐related genes and functional pathways, etc., in immune‐infiltrated cancer patients.

We discovered large sex differences of TMB in pan‐cancer and several individual cancer types. For example, the male patients in BLCA, KIRP, LIHC, LUAD, and SKCM showed higher TMB than female patients, and the female patients in GBM and STAD showed higher TMB than male patients (Fig. 2A). We validated these results in the independent validation data obtained from the cBioPortal and ICGC data portal (Table S3, Fig. S3). The limitation of our analysis is that the validation datasets which include cancer patients with the somatic mutation, expression, and clinical characteristics data are rare, and more cancer datasets must be obtained to validate our results in the future.

As TMB reflects the tumor antigenicity generated by somatic tumors, it may be more suitable to use patients with lower TMB for antigenicity‐enhancing strategies for tumor cells, such as radiation therapy and DNA‐damaging chemotherapy. Moreover, we discovered that female patients in HNSC, LUAD, and LUSC showed higher immune scores than male patients, and male patients in KIRP and SARC showed higher scores than female patients (Fig. S4). The stromal scores were higher in female patients than male patients in LUAD, and lower than male patients in SARC (Fig. S5). The immune and stromal scores reflect the immune microenvironment state, and the patients with lower immune and stromal scores may be more suitable for immune microenvironment‐enhancing strategies, such as hormone therapy and cytokine therapy, which may promote an immune response.

In a previous study, Wang et al. proposed that sex differences in immune response may be caused by differences in the expression of sex chromosome‐linked genes, in hormone levels, in developmental biology, etc.[6]. According to our analysis, LUAD, LUSC, HNSC, KIRP, SARC, and GBM were identified to be strongly sex‐biased in TME (Fig. 6). The potential mechanism of sex difference in TME may be the consequence of sex‐biased tumor molecular characteristics, such as somatic mutation, expression of immune checkpoint genes, which may perturb biological pathways and change prognostic biomarker performance.

## 5 Conclusions

Overall, the cancer immune system is complex, and may differ between male and female patients. Our study performed a comprehensive analysis to investigate sex‐based differences of tumor microenvironment‐related features across a broad range of cancer types. Our results thus provide a valuable starting point from which the role of sex can be explicitly considered in future ICB trials, identification of immune‐related biomarkers, and cancer immunotherapy.

## Conflict of interests

The authors declare that they have no competing interests.

## Author contributions

JH and YZ conceived and designed the study. YY and XL developed the methodology. JL and QW acquired the data. YS, JQ and YH analyzed the data and implemented the methodology. YY, YS and JW provided constructive discussions. JH and LC drafted the manuscript. All the authors read and agreed to the manuscript.

## Supporting information


**Fig. S1.** Flow chart of sample filtering.
**Fig. S2.** Box plots of TMB between male and female patients.
**Fig. S3.** Box plots of TMB between male and female patients in the validation sets.
**Fig. S4.** Box plots of immune scores between male and female patients.
**Fig. S5.** Box plots of stromal scores between male and female patients.
**Fig. S6.** Box plots of tumor purity between male and female patients.
**Fig. S7.** Dot plot of univariate hazard ratios (HR) and *P*‐values (Wald‐test) for each of the immune cells significantly associated with overall survival of patients (*P*‐value < 0.05) in male patient groups (119 male BLCA, 91 male COAD, 36 male GMB, 297 male HNSC, 154 male KIRC, 83 male KIRP, 27 male LAML, 29 male LIHC, 190 male LUAD, 309 male LUSC, 46 male PAAD, 68 male SARC, and 47 male THYM patients) and female patient groups (51 female BLCA, 71 female COAD, 11 female GMB, 76 female KIRC, 28 female LAML, 18 female LIHC, 228 female LUAD, 110 female LUSC, 71 female SARC, 83 female STAD, and 40 female PAAD patients).
**Fig. S8.** Kaplan–Meier survival curves of patients classified into high‐ and low‐risk groups using the male‐ and female‐specific prognostic score models in male and female cohorts.
**Fig. S9.** ROC curve for ICB therapy response prediction with TMB in male and female patients respectively.

**Table S1**. Summary of TCGA cancer types and immune infiltrated male and female patient samples used in the study.

**Table S2**. 24 different immune cell types in Bindea et al. publication and their marker genes.

**Table S3**. The validation datasets.

**Table S4**. Differential immune cells between male and female patients across 19 cancer types.

**Table S5**. Differential correlation of TMB with TME features (immune score, stromal score, tumor purity, and immune cell infiltration level) between male and female pan‐cancer patients.

**Table S6**. Differential correlation of TMB with immune cell infiltration level across different cancer types.

**Table S7**. Differentially expressed immune checkpoint genes between female and male (female/male) patients across 19 cancer types.

**Table S8**. Differential immune function pathways between female and male (female/male) patients across 19 cancer types.

**Table S9**. Hazard ratio (HR) and *P*‐value of overall survival for each immune cell in male and female patients, respectively, across 19 cancer types.

**Table S10**. Univariable and multivariable Cox regression analyses of male‐risk score/female‐risk score and clinicopathological factors in LUAD‐male/LUAD‐female cohort.

**Table S11**. Association of somatic mutation genes with patient sex bias on Chi‐square test in LUAD.

**Table S12**. Immune cells associated with sex‐biased mutation genes based on the multivariate logistic regression analysis in LUAD.

## Data Availability

The data that support the findings of this study are available on request from the corresponding author.
