# Supplementary material for: Pan‐cancer analysis reveals sex‐specific signatures in the tumor microenvironment
Source: Mol Oncol. 2022 Mar 12;16(11):2153–73. doi: 10.1002/1878-0261.13203 (PMC9168759; doi:10.1002/1878-0261.13203)
Supplement: Supplementary file 1 — Fig. S1. Flow chart of sample filtering. Fig. S2. Box plots of TMB between male and female patients. Fig. S3. Box plots of TMB between male and female patients in the validation sets. Fig. S4. Box plots of immune scores between male and female patients. Fig. S5. Box plots of stromal scores between male and female patients. Fig. S6. Box plots of tumor purity between male and female patients. Fig. S7. Dot plot of univariate hazard ratios (HR) and P‐values (Wald‐test) for each of the immune cells significantly associated with overall survival of patients (P‐value < 0.05) in male patient groups (119 male BLCA, 91 male COAD, 36 male GMB, 297 male HNSC, 154 male KIRC, 83 male KIRP, 27 male LAML, 29 male LIHC, 190 male LUAD, 309 male LUSC, 46 male PAAD, 68 male SARC, and 47 male THYM patients) and female patient groups (51 female BLCA, 71 female COAD, 11 female GMB, 76 female KIRC, 28 female LAML, 18 female LIHC, 228 female LUAD, 110 female LUSC, 71 female SARC, 83 female STAD, and 40 female PAAD patients). Fig. S8. Kaplan–Meier survival curves of patients classified into high‐ and low‐risk groups using the male‐ and female‐specific prognostic score models in male and female cohorts. Fig. S9. ROC curve for ICB therapy response prediction with TMB in male and female patients respectively. [file MOL2-16-2153-s011.pdf]

Supplementary material

Identification of Sex-specific Tumor Microenvironment Related Characterization and Prognostic Signatures

Inventory of Supplementary Information

1. Supplementary Text

2. Supplementary Figure S1-S9

Supplementary Text

Sex-specific immune cell prognostic markers

In LUAD, With Wald p<0.05 in univariate Cox proportional hazards model, we identified 8 significant immune cells were identified for female patients (Figure 7A), of which Tem cell, Tcm cell, iDC cell, Th17 cell, Tfh cell, mast cell, Macrophages cell are protective factors (HR<1) and Th2 cell are risk factors (HR>1). In contrast, 8 significant immune cells for male patients, of which Tem cell, iDC, pDC, Tfh cell, NK cell, T cell, and B cell are protective factors, and Th2 cell are risk factors. Although there are 4 significant cells are overlapped between male and female patients, their associated extent with survival is different between male and female patients. With the sex-specific cells, the male- and female- specific prognostic signatures were constructed respectively. For male- and female-specific signatures, we respectively calculated the risk score of signature for every male and female patient based on the cell infiltration level. Specifically, we derived two formulas to calculate the male- and female-specific risk score from the infiltration levels of significant male- and female-specific cells (p<0.05) weighted by the univariate Cox proportional hazard regression coefficient:

$$\textbf{female-risk scores} = -0.31\times \text{iDC} - 0.25\times \text{Macrophages} - 0.27\times \text{Mast cells} - 0.37\times \text{Tcm cells} - 0.38\times \text{Tem cells} - 0.29\times \text{Tfh cells} - 0.34\times \text{Th17 cells} + 0.35\times \text{Th2 cells} \quad (1)$$

$$\textbf{male-risk scores} = -0.10\times \text{B cells} - 0.31\times \text{iDC} - 0.24\times \text{NK cells} - 0.18\times \text{pDC} - 0.13\times \text{T cells} - 0.38\times \text{Tem cells} - 0.29\times \text{Tfh cells} + 0.35\times \text{Th2 cells} \quad (2)$$

We then used the Kaplan-Meier method and log-rank test to evaluate the power of classification of risk scores in male and female patients. According to the median of male- and female-risk scores, male and female patients were both classified into high-risk and low-risk groups (log-rank test p<1.0e-4 for female patients; p=1.8e-4 for male patients) (Figure 7B-C).

Furthermore, several other cancer types (such as LUSC and HNSC) also showed sex-biased features except LUAD (Figure 6). Thus, we also constructed the sex-specific risk score models in the strong sex-biased cancer types. For each of these cancer types, we respectively calculated male- and female-specific risk scores for each male and female patient based on the infiltration levels of significant male- and female-specific cells ($p<0.05$) weighted by the univariate Cox proportional hazard regression coefficient. The significant male- and female-specific cells for each cancer were listed in Supplementary Table S9. Then the Kaplan-Meier survival analysis and the log-rank test were performed to evaluate the power of classification of sex-specific risk scores in male and female patients in these cancers respectively. The Kaplan-Meier survival analysis showed that patients in the low-risk group had longer OS than those in the high-risk group, although this difference was not statistically significant in SARC-Female, KIRP-Female, LUSC-Female cohorts (Supplementary Figure S8A-I). Additionally, we did not find the female-specific risk scoring model in the HNSC-Female cohort as there were no significant prognostic associated cells. These results validated that the sex-specific risk score models could classify patients into high-risk and low-risk groups for male and female patients in most of these sex-biased cancer types.

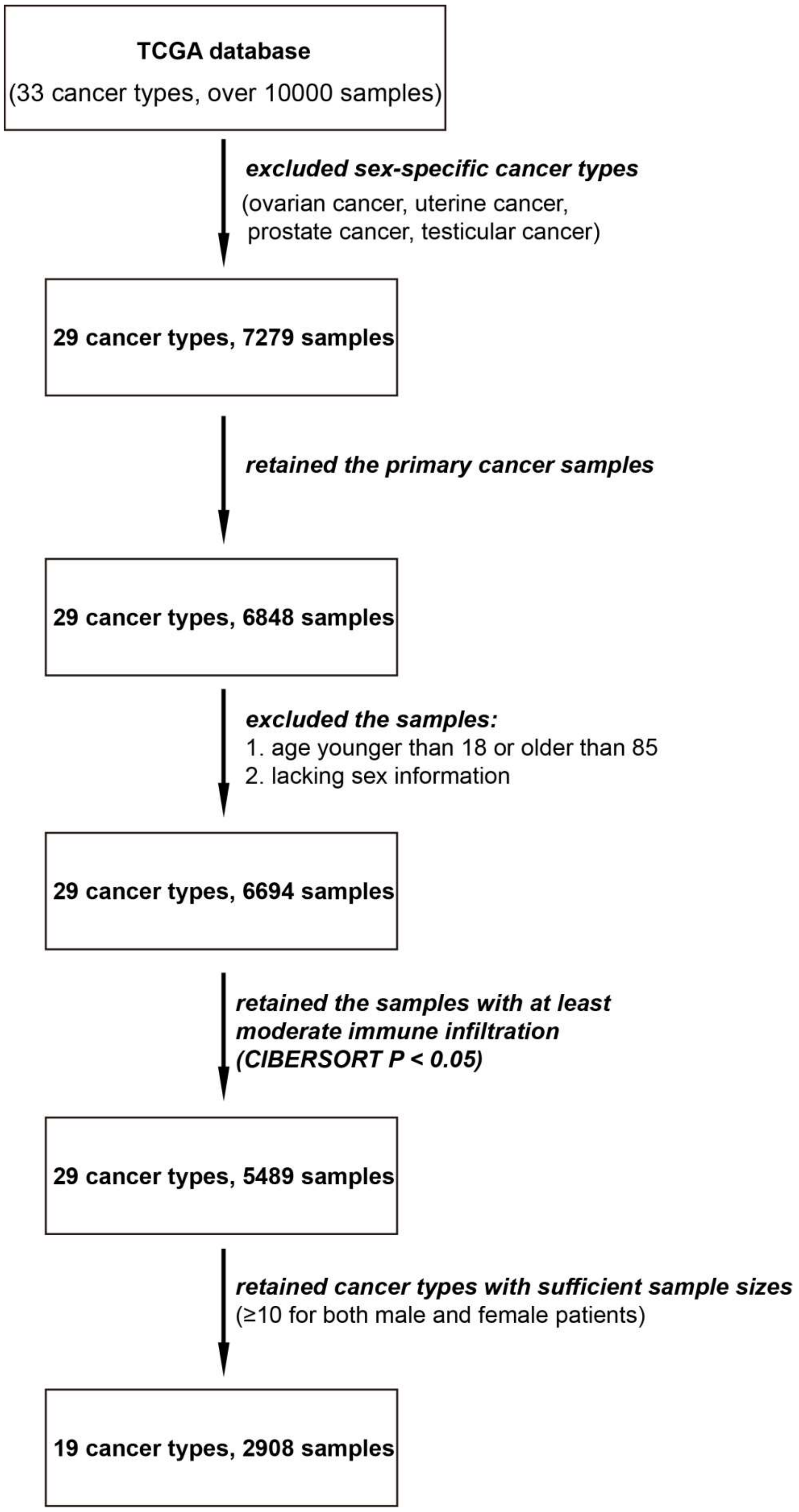

Supplementary Figure S1. Flow chart of sample filtering.

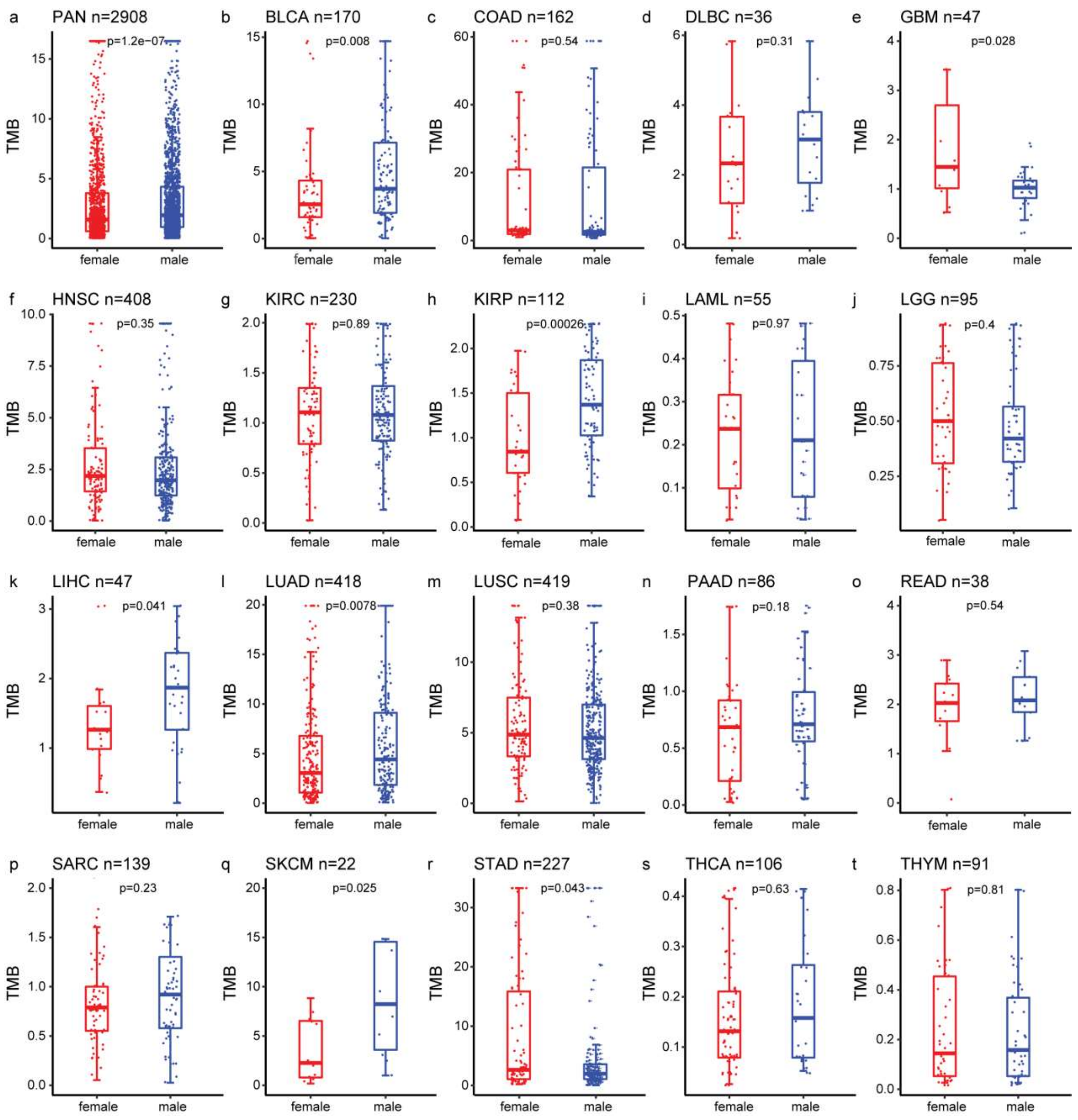

Supplementary Figure S2. Box plots of TMB between male and female patients. (a) Pan-cancer patient cohorts (1,771 male and 1,137 female patients). (b) BLCA patient cohort (119 male and 51 female patients). (c) COAD patient cohort (91 male and 71 female patients). (d) DLBC patient cohort (16 male and 20 female patients). (e) GMB patient cohort (36 male and 11 female patients). (f) HNSC patient cohort (297 male and 111 female patients). (g) KIRC patient cohort (154 male and 76 female patients). (h) KIRP patient cohort (83 male and 29 female patients). (i) LAML patient cohort (27 male and 28 female patients). (j) LGG patient cohort (55 male and 40 female patients). (k) LIHC patient cohort (29 male and 18 female patients). (l) LUAD patient cohort (190 male and 228 female patients). (m) LUSC patient cohort (309 male and 110 female patients). (n) PAAD patient cohort (46 male and 40 female patients). (o) READ patient cohort (21 male and 17 female patients). (p) SARC patient cohort (68 male and 71 female patients). (q) SKCM patient cohort (10 male and 12 female patients). (r) STAD patient cohort (144 male and 83 female patients). (s) THCA patient cohort (29 male and 77 female patients). (t) THYM patient cohort (47 male and 44 female patients). The top and bottom error bars indicate the 90th and 10th percentiles of TMB. The statistical significance (p-value) was determined by the Wilcoxon rank-sum test.

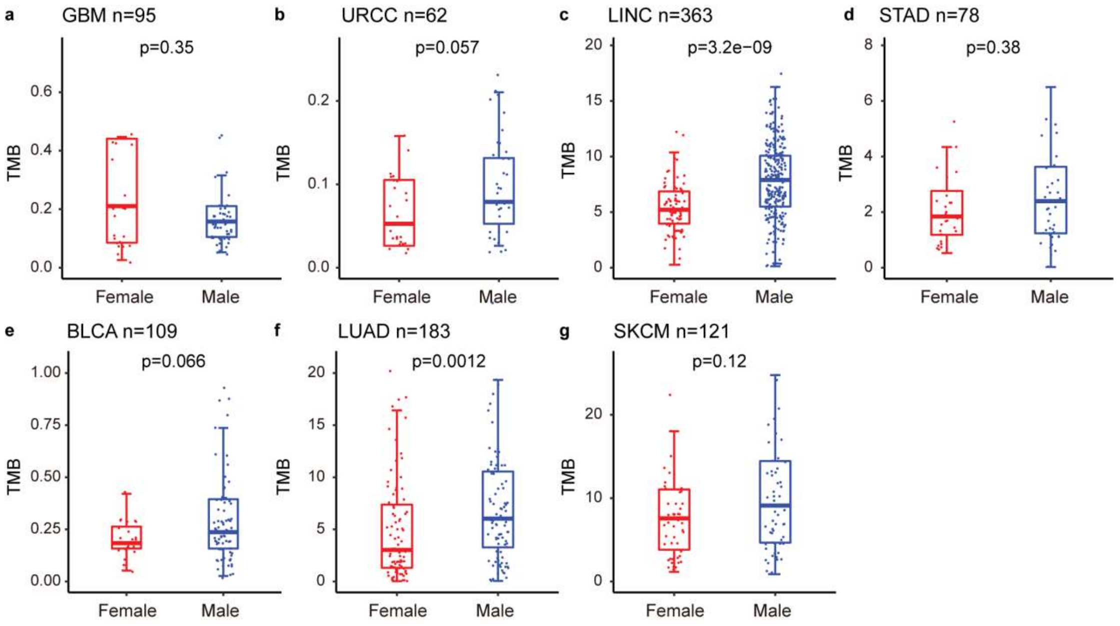

Supplementary Figure S3. Box plots of TMB between male and female patients in the validation sets. (a) GBM patient cohort (57 male and 38 female patients). (b) URCC patient cohort (34 male and 28 female patients). (c) LINC patient cohort (277 male and 86 female patients). (d) STAD patient cohort (45 male and 33 female patients). (e) BLCA patient cohort (82 male and 27 female patients). (f) LUAD patient cohort (95 male and 88 female patients). (g) SKCM patient cohort (67 male and 54 female patients). The top and bottom error bars indicate the 90th and 10th percentiles of TMB. The statistical significance (p-value) was determined by the Wilcoxon rank-sum test.

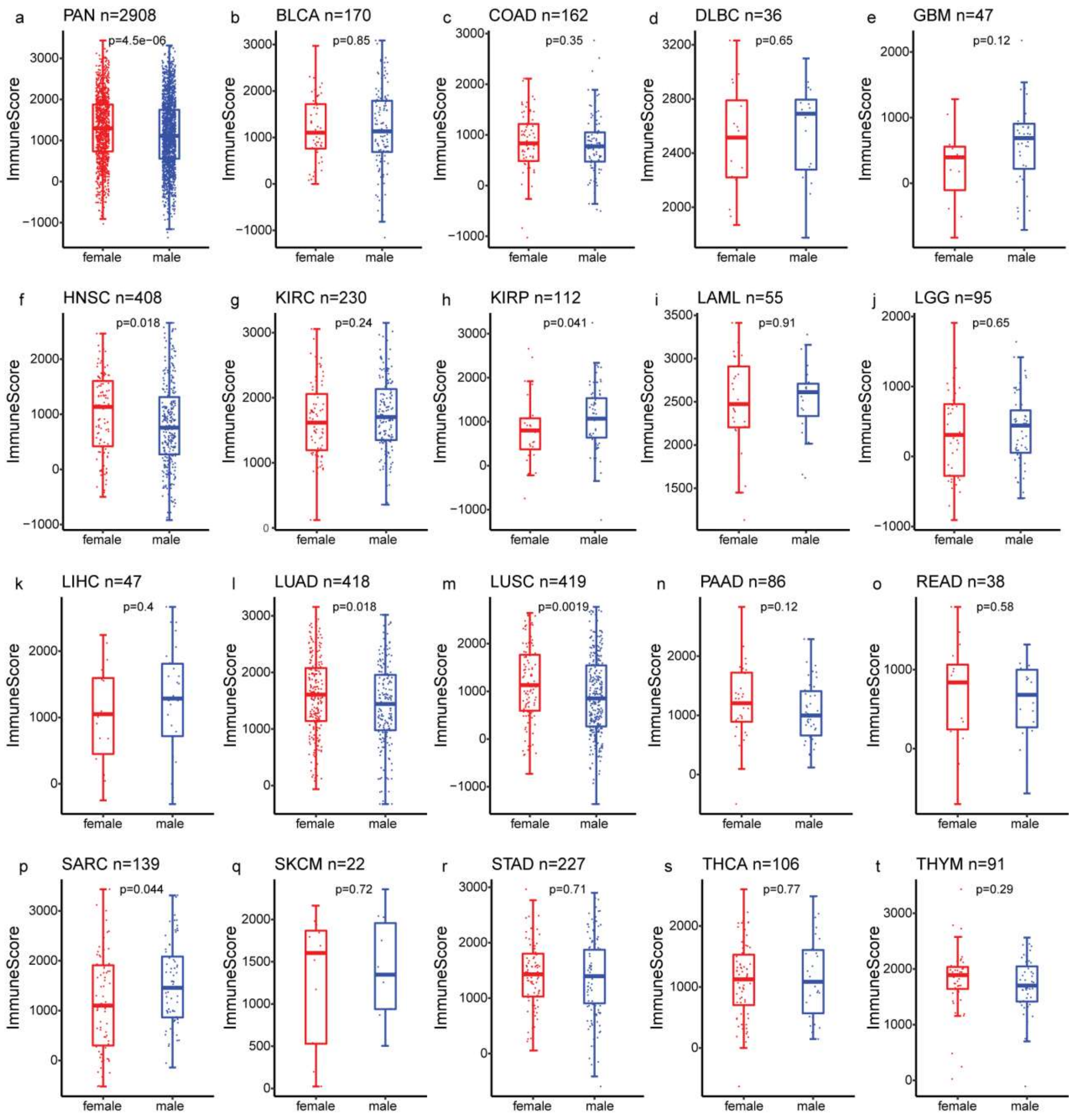

Supplementary Figure S4. Box plots of immune scores between male and female patients. (a) Pan-cancer patient cohorts (1,771 male and 1,137 female patients). (b) BLCA patient cohort (119 male and 51 female patients). (c) COAD patient cohort (91 male and 71 female patients). (d) DLBC patient cohort (16 male and 20 female patients). (e) GMB patient cohort (36 male and 11 female patients). (f) HNSC patient cohort (297 male and 111 female patients). (g) KIRC patient cohort (154 male and 76 female patients). (h) KIRP patient cohort (83 male and 29 female patients). (i) LAML patient cohort (27 male and 28 female patients). (j) LGG patient cohort (55 male and 40 female patients). (k) LIHC patient cohort (29 male and 18 female patients). (l) LUAD patient cohort (190 male and 228 female patients). (m) LUSC patient cohort (309 male and 110 female patients). (n) PAAD patient cohort (46 male and 40 female patients). (o) READ patient cohort (21 male and 17 female patients). (p) SARC patient cohort (68 male and 71 female patients). (q) SKCM patient cohort (10 male and 12 female patients). (r) STAD patient cohort (144 male and 83 female patients). (s) THCA patient cohort (29 male and 77 female patients). (t) THYM patient cohort (47 male and 44 female patients). The top and bottom error bars indicate the 90th and 10th percentiles of immune scores. The statistical significance (p-value) was determined by the Wilcoxon rank-sum test.

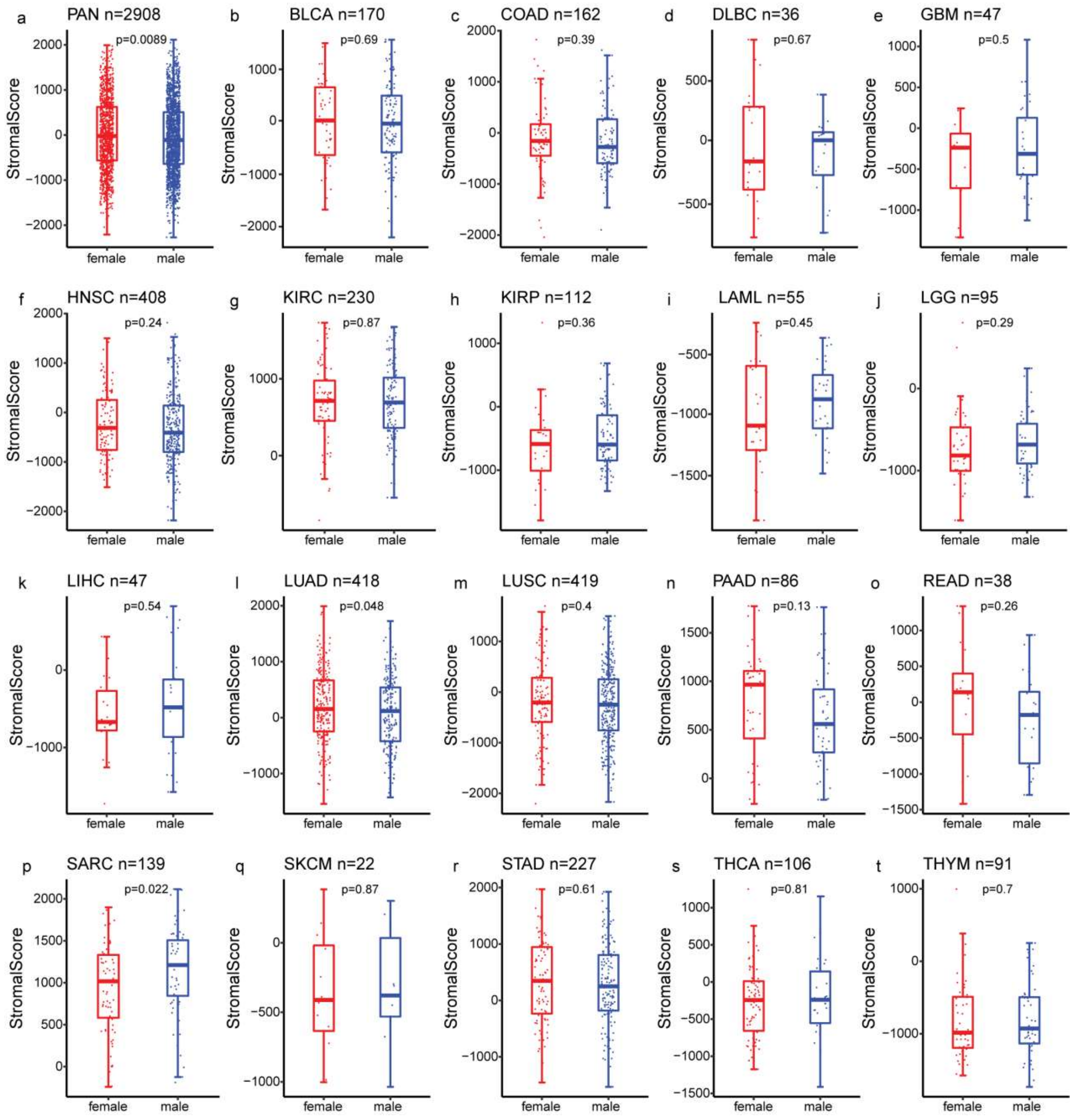

Supplementary Figure S5. Box plots of stromal scores between male and female patients. (a) Pan-cancer patient cohorts (1,771 male and 1,137 female patients). (b) BLCA patient cohort (119 male and 51 female patients). (c) COAD patient cohort (91 male and 71 female patients). (d) DLBC patient cohort (16 male and 20 female patients). (e) GMB patient cohort (36 male and 11 female patients). (f) HNSC patient cohort (297 male and 111 female patients). (g) KIRC patient cohort (154 male and 76 female patients). (h) KIRP patient cohort (83 male and 29 female patients). (i) LAML patient cohort (27 male and 28 female patients). (j) LGG patient cohort (55 male and 40 female patients). (k) LIHC patient cohort (29 male and 18 female patients). (l) LUAD patient cohort (190 male and 228 female patients). (m) LUSC patient cohort (309 male and 110 female patients). (n) PAAD patient cohort (46 male and 40 female patients). (o) READ patient cohort (21 male and 17 female patients). (p) SARC patient cohort (68 male and 71 female patients). (q) SKCM patient cohort (10 male and 12 female patients). (r) STAD patient cohort (144 male and 83 female patients). (s) THCA patient cohort (29 male and 77 female patients). (t) THYM patient cohort (47 male and 44 female patients). The top and bottom error bars indicate the

90th and 10th percentiles of stromal scores. The statistical significance (p-value) was determined by the Wilcoxon rank-sum test.

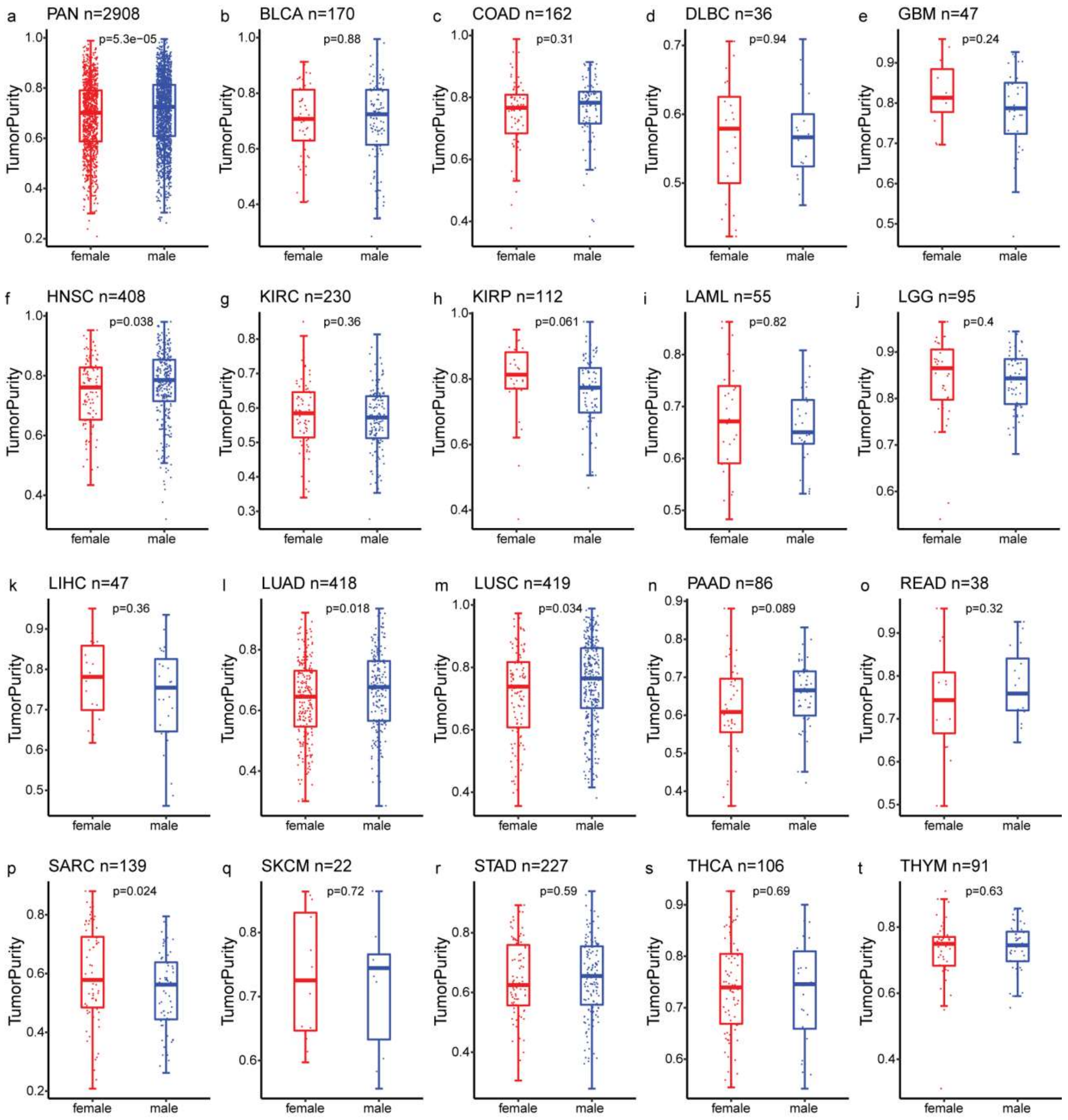

Supplementary Figure S6. Box plots of tumor purity between male and female patients. (a) Pan-cancer patient cohorts (1,771 male and 1,137 female patients). (b) BLCA patient cohort (119 male and 51 female patients). (c) COAD patient cohort (91 male and 71 female patients). (d) DLBC patient cohort (16 male and 20 female patients). (e) GMB patient cohort (36 male and 11 female patients). (f) HNSC patient cohort (297 male and 111 female patients). (g) KIRC patient cohort (154 male and 76 female patients). (h) KIRP patient cohort (83 male and 29 female patients). (i) LAML patient cohort (27 male and 28 female patients). (j) LGG patient cohort (55 male and 40 female patients). (k) LIHC patient cohort (29 male and 18 female patients). (l) LUAD patient cohort (190 male and 228 female patients). (m) LUSC patient cohort (309 male and 110 female patients). (n) PAAD patient cohort (46 male and 40 female patients). (o) READ patient cohort (21 male and 17 female patients). (p) SARC patient cohort (68 male and 71 female patients). (q) SKCM patient cohort (10 male and 12 female patients). (r) STAD patient cohort

(144 male and 83 female patients). (s) THCA patient cohort (29 male and 77 female patients). (t) THYM patient cohort (47 male and 44 female patients). The top and bottom error bars indicate the 90th and 10th percentiles of tumor purity. The statistical significance (p-value) was determined by the Wilcoxon rank-sum test.

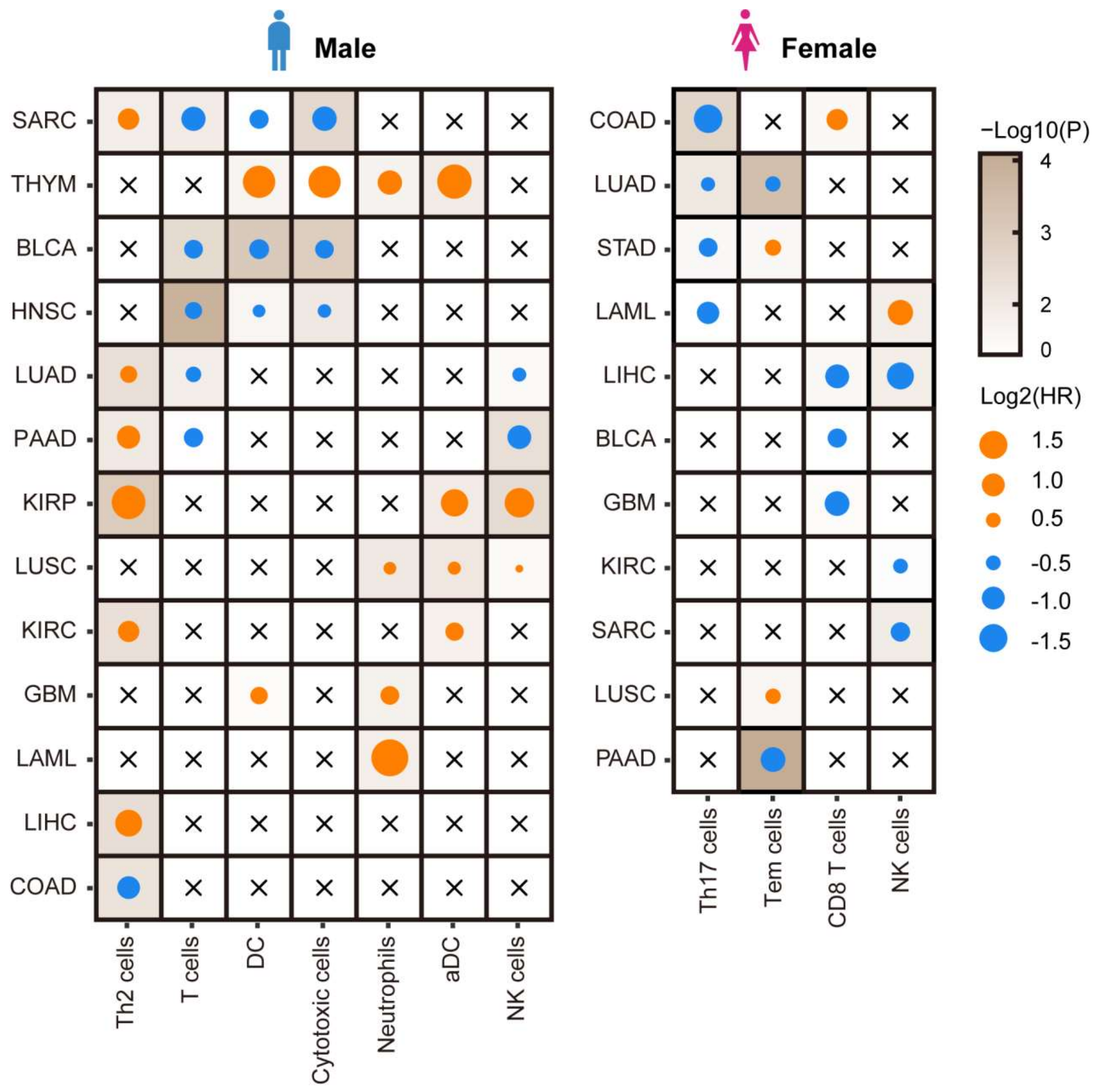

Supplementary Figure S7**.** Dot plot of univariate hazard ratios (HRs) and p-values (Wald-test) for each of the immune cells significantly associated with overall survival of patients (p-value < 0.05) in male patient groups (119 male BLCA, 91 male COAD, 36 male GMB, 297 male HNSC, 154 male KIRC, 83 male KIRP, 27 male LAML, 29 male LIHC, 190 male LUAD, 309 male LUSC, 46 male PAAD, 68 male SARC, and 47 male THYM patients) and female patient groups (51 female BLCA, 71 female COAD, 11 female GMB, 76 female KIRC, 28 female LAML, 18 female LIHC, 228 female LUAD, 110 female LUSC, 71 female SARC, 83 female STAD, and 40 female PAAD patients) respectively. The significant immune cells associated with at least four cancer types are retained in male and female patient groups respectively.

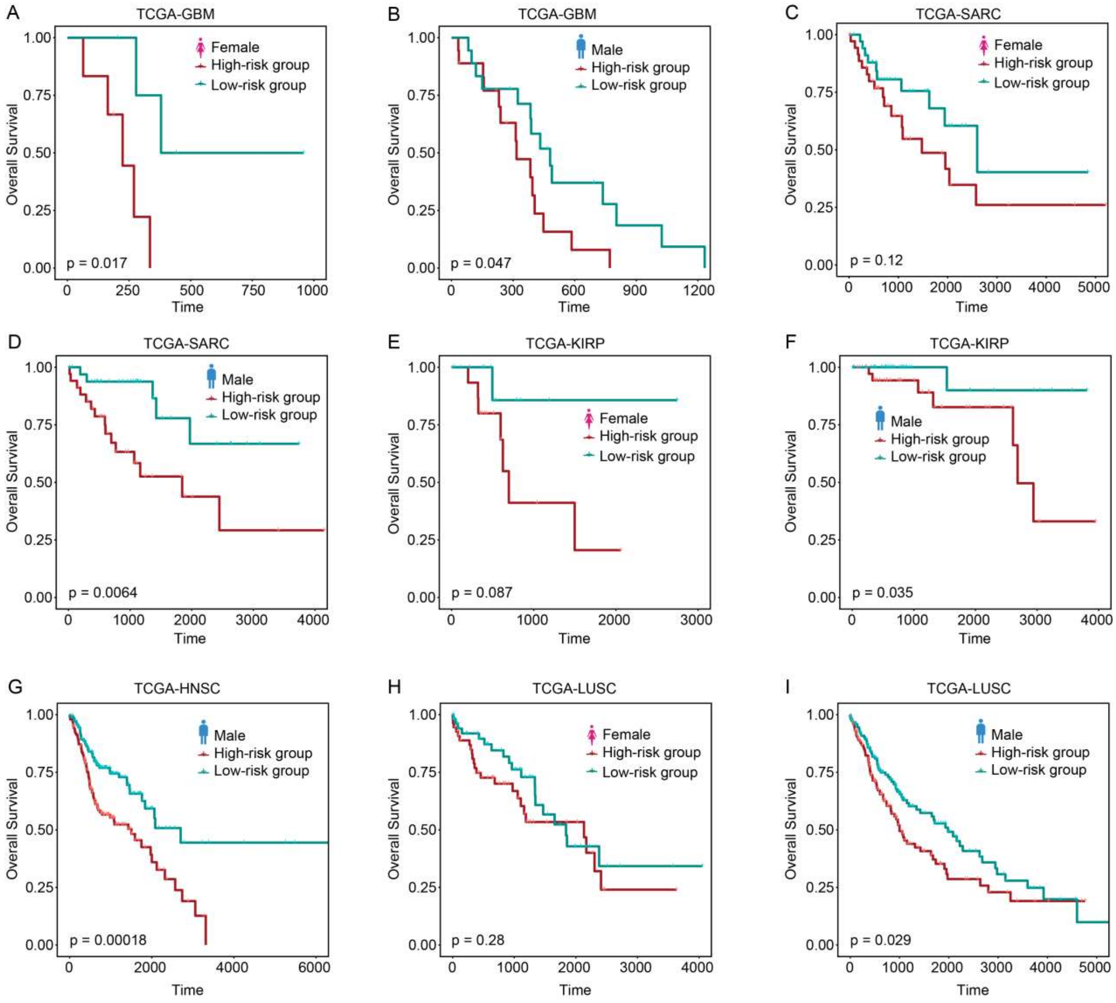

Supplementary Figure S8. Kaplan-Meier survival curves of patients classified into high- and low-risk groups using the male- and female-specific prognostic score models in male and female cohorts. (A) GBM-female patient cohort (11 samples). (B) GBM-male patient cohort (36 samples). (C) SARC-female patient cohort (71 samples). (D) SARC-male patient cohort (68 samples). (E) KIRP-female patient cohort (29 samples). (F) KIRP-male patient cohort (83 samples). (G) HNSC-male patient cohort (297 samples). (H) LUSC-female patient cohort (110 samples). (I) LUSC-male patient cohort (309 samples). The statistical significance (p-value) was determined by the log-rank test.

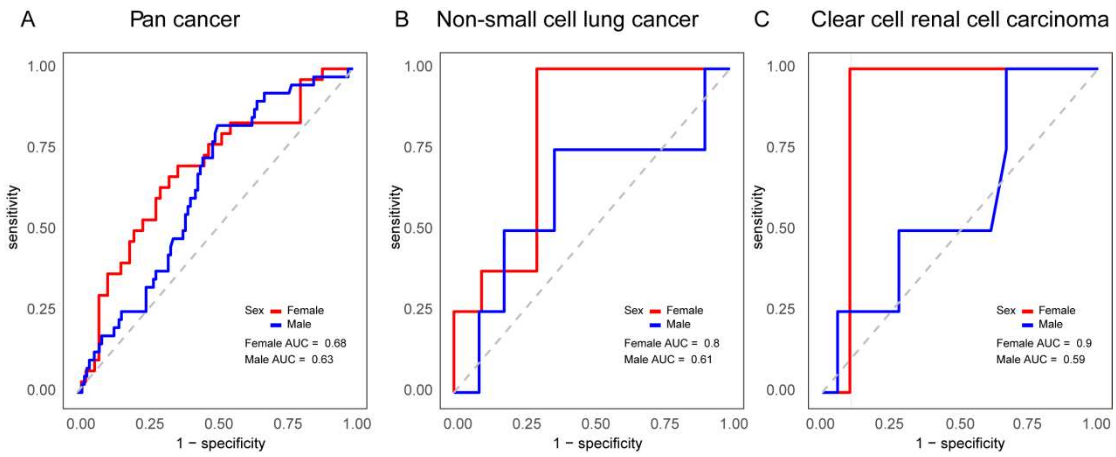

Supplementary Figure S9. ROC curve for ICB therapy response prediction with TMB in male and female patients respectively. (A) Pan-cancer patient cohorts (154 male and 95 female patients). (B) Non-small cell lung cancer patient cohorts (16 male and 18 female patients), and (C) Clear cell renal cell carcinoma patient cohorts (22 male and 13 female patients).
