# Supplementary material for: Pan‐cancer analysis reveals sex‐specific signatures in the tumor microenvironment
Source: Mol Oncol. 2022 Mar 12;16(11):2153–73. doi: 10.1002/1878-0261.13203 (PMC9168759; doi:10.1002/1878-0261.13203)
Supplement: Supplementary file 2 — Table S1. Summary of TCGA cancer types and immune infiltrated male and female patient samples used in the study. [file MOL2-16-2153-s003.docx]

Supplementary Table S1. Summary of TCGA cancer types and immune infiltrated male and female patient samples used in the study.

| Cancer abbreviation | Full name | Female | Male | Total |
| --- | --- | --- | --- | --- |
| BLCA | Bladder Urothelial Carcinoma | 51 | 119 | 170 |
| COAD | Colon adenocarcinoma | 71 | 91 | 162 |
| DLBC | Lymphoid Neoplasm Diffuse Large B-cell Lymphoma | 20 | 16 | 36 |
| GBM | Glioblastomamultiforme | 11 | 36 | 47 |
| HNSC | Head and Neck squamous cell carcinoma | 111 | 297 | 408 |
| KIRC | Kidney renal clear cell carcinoma | 76 | 154 | 230 |
| KIRP | Kidney renal papillary cell carcinoma | 29 | 83 | 112 |
| LAML | Acute Myeloid Leukemia | 28 | 27 | 55 |
| LGG | Brain Lower Grade Glioma | 40 | 55 | 95 |
| LIHC | Liver hepatocellular carcinoma | 18 | 29 | 47 |
| LUAD | Lung adenocarcinoma | 228 | 190 | 418 |
| LUSC | Lung squamous cell carcinoma | 110 | 309 | 419 |
| PAAD | Pancreatic adenocarcinoma | 40 | 46 | 86 |
| READ | Rectum adenocarcinoma | 17 | 21 | 38 |
| SARC | Sarcoma | 71 | 68 | 139 |
| SKCM | Skin Cutaneous Melanoma | 12 | 10 | 22 |
| STAD | Stomach adenocarcinoma | 83 | 144 | 227 |
| THCA | Thyroid carcinoma | 77 | 29 | 106 |
| THYM | Thymoma | 44 | 47 | 91 |
| Total |  | 1137 | 1771 | 2908 |
