# Supplementary material for: Pan‐cancer analysis reveals sex‐specific signatures in the tumor microenvironment
Source: Mol Oncol. 2022 Mar 12;16(11):2153–73. doi: 10.1002/1878-0261.13203 (PMC9168759; doi:10.1002/1878-0261.13203)
Supplement: Supplementary file 11 — Table S10. Univariable and multivariable Cox regression analyses of male‐risk score/female‐risk score and clinicopathological factors in LUAD‐male/LUAD‐female cohort. [file MOL2-16-2153-s005.docx]

|  | **Univariate analysis** | | |  | | **Multivariate analysis** | | |
| --- | --- | --- | --- | --- | --- | --- | --- | --- |
|  | **HR** | **95%CI** | **P value** | |  | **HR** | **95%CI** | **P value** |
| **LUAD-male cohor**t |  |  |  | |  |  |  |  |
| Male-risk score  (low vs high) | 0.40 | 0.24-0.66 | <0.01 | |  | 0.42 | 0.25-0.70 | <0.01 |
| Age (≥65 vs <65) | 1.20 | 0.73-1.90 | 0.48 | |  | 1.31 | 0.81-2.14 | 0.27 |
| Stage (III/IV vs I/II) | 2.50 | 1.50-4.20 | <0.01 | |  | 2.17 | 1.29-3.65 | <0.01 |
| **LUAD-female cohort** |  |  |  | |  |  |  |  |
| Female-risk score  (low vs high) | 0.38 | 0.24-0.62 | <0.01 | |  | 0.44 | 0.27-0.72 | <0.01 |
| Age (≥65 vs <65) | 1.10 | 0.68-1.70 | 0.77 | |  | 1.12 | 0.71-1.77 | 0.62 |
| Stage (III/IV vs I/II) | 2.60 | 1.60-4.20 | <0.01 | |  | 2.16 | 1.32-3.52 | <0.01 |

**Supplementary Table S10**. Univariable and multivariable Cox regression analyses of male-risk score/female-risk score and clinicopathological factors in LUAD-male/LUAD-female cohort.
